# Mechanistic modeling quantifies the influence of tumor growth kinetics on the response to anti-angiogenic treatment

**DOI:** 10.1371/journal.pcbi.1005874

**Published:** 2017-12-21

**Authors:** Thomas D. Gaddy, Qianhui Wu, Alyssa D. Arnheim, Stacey D. Finley

**Affiliations:** 1 Department of Chemical Engineering and Materials Science, University of Southern California, Los Angeles, California, United States of America; 2 Department of Biomedical Engineering, University of Southern California, Los Angeles, California, United States of America; 3 Department of Biomedical Engineering, Boston University, Boston, Massachusetts, United States of America; Johns Hopkins University, UNITED STATES

## Abstract

Tumors exploit angiogenesis, the formation of new blood vessels from pre-existing vasculature, in order to obtain nutrients required for continued growth and proliferation. Targeting factors that regulate angiogenesis, including the potent promoter vascular endothelial growth factor (VEGF), is therefore an attractive strategy for inhibiting tumor growth. Computational modeling can be used to identify tumor-specific properties that influence the response to anti-angiogenic strategies. Here, we build on our previous systems biology model of VEGF transport and kinetics in tumor-bearing mice to include a tumor compartment whose volume depends on the “angiogenic signal” produced when VEGF binds to its receptors on tumor endothelial cells. We trained and validated the model using published *in vivo* measurements of xenograft tumor volume, producing a model that accurately predicts the tumor’s response to anti-angiogenic treatment. We applied the model to investigate how tumor growth kinetics influence the response to anti-angiogenic treatment targeting VEGF. Based on multivariate regression analysis, we found that certain intrinsic kinetic parameters that characterize the growth of tumors could successfully predict response to anti-VEGF treatment, the reduction in tumor volume. Lastly, we use the trained model to predict the response to anti-VEGF therapy for tumors expressing different levels of VEGF receptors. The model predicts that certain tumors are more sensitive to treatment than others, and the response to treatment shows a nonlinear dependence on the VEGF receptor expression. Overall, this model is a useful tool for predicting how tumors will respond to anti-VEGF treatment, and it complements pre-clinical *in vivo* mouse studies.

## Author summary

One hallmark of cancer is angiogenesis, the formation of new blood capillaries from pre-existing vessels. Angiogenesis promotes tumor growth by enabling the tumor to obtain oxygen and nutrients from the surrounding microenvironment. Cancer drugs that inhibit angiogenesis ("anti-angiogenic therapies") have focused on inhibiting proteins that promote the growth of new blood vessels. The response to anti-angiogenic therapy is highly variable, and some tumors do not respond at all. Therefore, identifying a biomarker that predicts how specific tumors will respond would be extremely valuable. This work uses a computational model of tumor-bearing mice to investigate the response to anti-angiogenic treatment that targets the potent promoter of angiogenesis, vascular endothelial growth factor (VEGF), and how the response is influenced by tumor growth kinetics. We show that certain properties of tumor growth can be used to predict how much the tumor volume will be reduced upon administration of an anti-VEGF drug. This work identifies tumor growth parameters that may be reliable biomarkers for predicting how tumors will respond to anti-VEGF therapy. Our computational model generates novel, testable hypotheses and nicely complements pre-clinical studies of anti-angiogenic therapeutics.

## Introduction

Angiogenesis is the formation of new blood vessels from pre-existing vasculature and is important in both physiological and pathological conditions. Numerous promoters and inhibitors regulate angiogenesis. One key promoter of angiogenesis is the vascular endothelial growth factor-A (VEGF-A), which has been extensively studied and is a member of a family of pro-angiogenic factors that includes five ligands: VEGF-A, VEGF-B, VEGF-C, VEGF-D, and placental growth factor (PlGF). VEGF-A (or simply, VEGF) promotes angiogenesis by binding to its receptors VEGFR1 and VEGFR2 and recruiting co-receptors called neuropilins (NRP1 and NRP2). The VEGF receptors and co-receptors are expressed on many different cell types, including endothelial cells (ECs), cancer cells, neurons, and muscle fibers [1]. Together, VEGF and its receptors and co-receptors initiate the intracellular signaling necessary to promote vessel sprouting, and ultimately, the formation of fully matured and functional vessels. The new vasculature formed following VEGF signaling enables delivery of oxygen and nutrients and facilitates removal of waste products [2].

Regulating angiogenesis presents an attractive treatment strategy for diseases characterized by either insufficient or excessive vascularization. In the context of excessive vascularization seen in many types of cancer, inhibiting angiogenesis can decrease tumor growth. Anti-angiogenic treatment targeting tumor vascularization is a particular focus area within cancer research [3]. One anti-angiogenic drug is bevacizumab, a recombinant monoclonal antibody that neutralizes VEGF (an “anti-VEGF” drug). Bevacizumab is approved as a monotherapy or in combination with chemotherapy for several cancers, including metastatic colorectal cancer, non-small cell lung cancer, and metastatic cervical cancer [4]. In 2008, the drug gained accelerated approval for treatment of metastatic breast cancer (mBC) through the US Food and Drug Administration (FDA), based on evidence from pre-clinical studies and early phase clinical trials. Though initial clinical trials showed that bevacizumab improved progression-free survival (PFS), subsequent results revealed that bevacizumab failed to improve overall survival (OS) in a wide range of patients and that the drug elicited significant adverse side effects [5]. Consequently, the FDA revoked its approval for the use of bevacizumab for mBC in late 2011 [6].

The case of bevacizumab illustrates that although anti-angiogenic therapy can be effective, not all patients or cancer types respond to the treatment. This underscores the need for biomarkers that can help select patients who are likely to respond to anti-angiogenic treatment. Numerous studies have sought to identify biomarkers for anti-angiogenic treatment. Biomarkers can be used to determine which tumors will respond prior to any treatment being given (“predictive”), or to evaluate efficacy following treatment (“prognostic”) [7]. Biomarkers can also be used to determine optimal doses, to design combination therapies, and to indicate resistance to therapies [8]. The concentration range of circulating angiogenic factors (CAFs), and VEGF in particular, is one possible predictor of the response to anti-angiogenic therapy [7]. Alternatively, expression of angiogenic receptors such as NRP1 and VEGFR1 on tumor cells, in the tumor interstitial space, or in plasma can serve as biomarker candidates [5,9]. Unfortunately, though some of these candidates are promising, a marker that predicts bevacizumab treatment outcome has not yet been validated [5,7]. In fact, relying on the concentrations of CAFs has produced inconclusive and inconsistent results [7,8,10]. Tumor growth kinetics have also been investigated as prognostic biomarkers of the response to anti-angiogenic treatment [11–15]. The most recent studies take advantage of improved imaging technology that can assess tumor volume, rather than only providing two-dimensional information [11]. The imaging analyses show that tumor growth kinetics may be a reliable indicator of treatment efficacy and are in good agreement with standardized approaches for assessing response treatment. However, utilizing tumor growth kinetics as a predictive biomarker has not been extensively studied.

Mouse models present a useful platform for cancer research, including biomarker discovery. Despite differences in the mouse and human anatomy and immune system, pre-clinical mouse studies are useful in understanding human cancer progression and response to therapy [16]. Advances in molecular biology techniques have generated relevant mouse models (i.e., patient-derived tumor models and genetically engineered models). These mouse models enable biomarker discovery for early detection of cancer [17], to identify non-responders to a particular treatment [18], and to classify tumors as being drug-sensitive or drug-resistant [19]. Excitingly, computational analyses are being combined with pre-clinical models to identify biomarkers for early detection and progression [17,19].

There is a substantial and productive history of applying computational modeling to study cancer at multiple scales, from initiation through metastasis [20–22]. The model predictions provide testable hypotheses that have been experimentally and clinically validated. Given the multiple cell types, molecular species and signaling pathways involved in angiogenesis, systems biology approaches are used to understand the dynamic ligand-receptor interactions that mediate angiogenesis and tumor growth. Systems biology studies how individual components of biological systems give rise to the function and behavior of the system and aims to predict this behavior by combining quantitative experimental techniques and computational models [23]. Our previous work and the work of others demonstrate that mathematical models complement pre-clinical and clinical angiogenesis research [8,24]. These models have been used to identify prognostic biomarkers that can predict which patients will benefit from anti-angiogenic therapies [24–26].

In this work, we use a computational systems biology model to investigate the utility of tumor growth kinetics in predicting response to anti-VEGF treatment. We make use of quantitative measurements from pre-clinical mouse studies and use those data to train the computational model. This work builds upon our previous computational model of VEGF distribution and kinetics in tumor-bearing mice [27] by changing the dynamic tumor volume to be dependent on the pro-angiogenic complexes involving VEGF-bound receptors (the “angiogenic signal”). This new element of the computational model allows us to simulate anti-VEGF treatment and predict the effect of the treatment on tumor volume. We apply the new model to identify conditions and characteristics of tumor growth that may be predictive of a favorable response to anti-angiogenic treatment. Our work contributes to the identification of validated biomarkers that could be used to determine tumors that are sensitive to anti-angiogenic treatment.

## Results

### Model construction

We have previously developed compartmental models to investigate the kinetics and transport of VEGF, a key regulator of angiogenesis [28–32]. In our previous computational model, the dynamic tumor volume was given by an exponential function and was not linked to the concentrations of pro-angiogenic species. We now address this limitation of our previous work. Specifically, we expand our previous computational model of VEGF distribution in tumor-bearing mice [28] to incorporate the effect of VEGF on tumor growth. Having the dynamic tumor volume be a function of the concentration of VEGF bound to receptors on tumor endothelial cells is a significant improvement and generates a more physiologically relevant computational tool to investigate anti-angiogenic treatment strategies.

Details regarding the model structure and molecular species are provided in the Methods Section. Here, we detail the equation for tumor growth. Tumor growth is given by an adapted Gompertz model focusing on the exponential and linear phases of the tumor, as previously described [8,33]. Thus, the differential equation for the tumor volume (termed “Tumor Growth Model 1”) is:

$$\frac{dV(t)}{dt}=\frac{k_{0}*V(t)}{{\left[1+{\left(\frac{k_{0}}{k_{1}}*V(t)\right)}^{\psi }\right]}^{\frac{1}{\psi }}}∙\left(1-\frac{{Ang}_{0}-Ang(t)}{{Ang}_{0}}\right)$$ (1A)

We note that Eq (1A) simplifies to:

$$\frac{dV(t)}{dt}=\frac{k_{0}*V(t)}{{\left[1+{\left(\frac{k_{0}}{k_{1}}*V(t)\right)}^{\psi }\right]}^{\frac{1}{\psi }}}∙\left(\frac{Ang(t)}{{Ang}_{0}}\right)$$ (1B)

Here, *V(t)* is the tumor volume in cm^3^ at time *t*, *k*_*0*_ and *k*_*1*_ are parameters describing the rate of exponential and linear growth, respectively. The units of *k*_*0*_ and *k*_*1*_ are s^-1^ and cm^3^ tissue/s, respectively. The *ѱ* parameter represents the transition from exponential to linear tumor growth and is unitless. The *Ang*_*0*_ parameter represents the basal angiogenic signal (at time *t* = 0), and *Ang(t)* is the angiogenic signal at time *t*. The value of *Ang* at any time is calculated as the total concentration of pro-angiogenic VEGF-receptor complexes on tumor endothelial cells. This includes VEGFR1 and VEGFR2 bound to either mouse or human VEGF isoforms, with or without the NRP1 co-receptor. Thus, *Ang(t)* and *Ang*_*0*_ have units of concentration (mol/cm^3^ tissue). The values of the tumor growth parameters were estimated by fitting the model to experimental data, as described in the following section.

### Model fitting

We fit the model to control data from published experimental datasets quantifying tumor volume in mice bearing MDA-MB-231 xenograft tumors without any anti-VEGF treatment [34–38]. The raw data used for fitting (extracted from published references; see Methods for details) are provided in the S1 Table. Although all of the datasets were generated using the same breast cancer cell line, tumor growth is variable in each case, with the final tumor volume ranging from 0.8–2.5 cm^3^. Additionally, the tumors follow different growth profiles (S2 Table). These differences in the final volume and growth kinetics can be attributed to differences in the experimental methods from each dataset, including the mouse strain used, number of tumor cells injected, and the location of the tumor cell injection. Finally, the researchers quantify tumor volume using different equations. We aim to identify tumor growth kinetic parameters for individual tumors; therefore, we fit each dataset individually in the parameter estimation. This allows us to determine tumor-specific growth parameters, even for mice with the same type of tumor.

**Fig 1:**
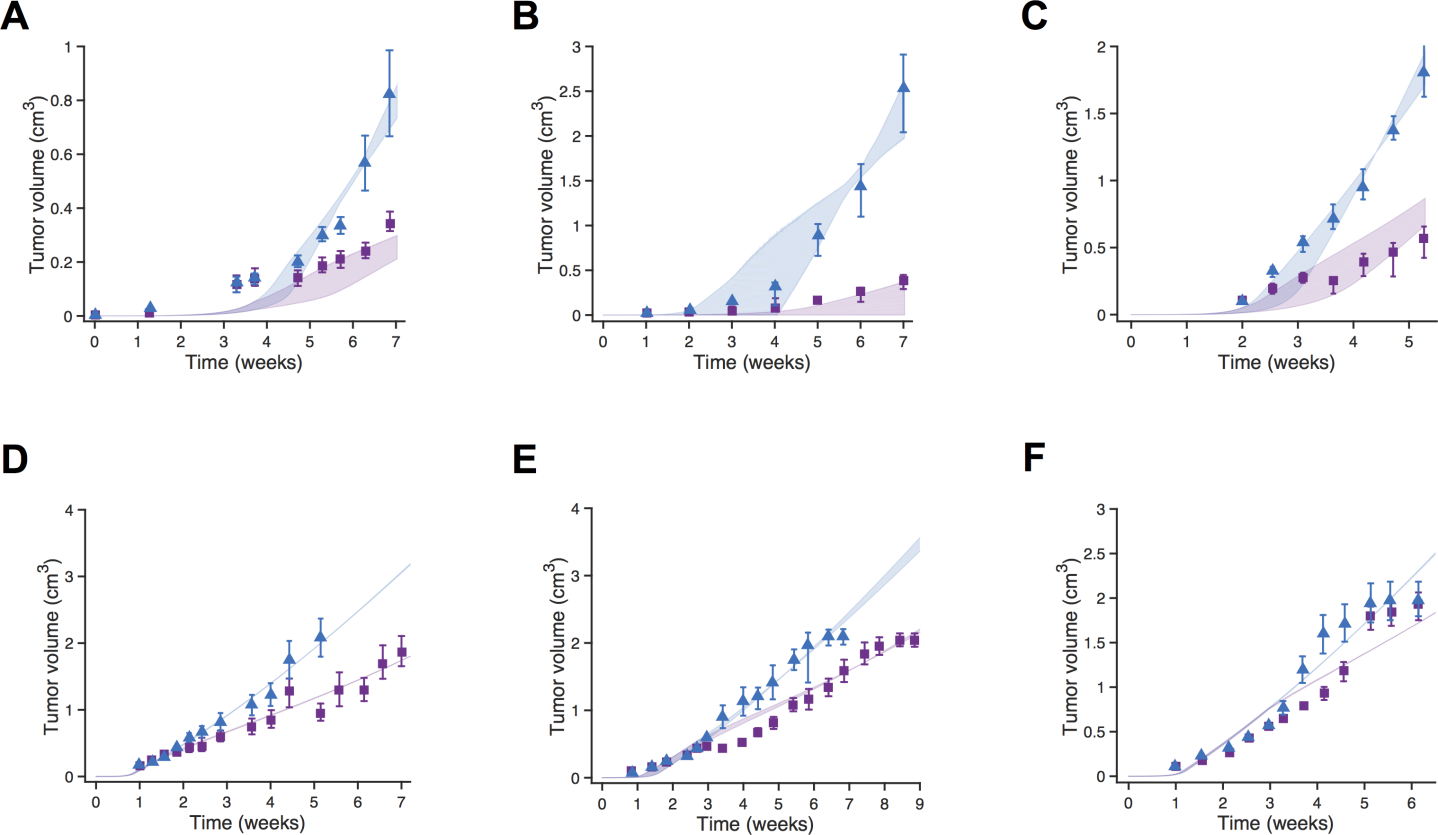
Model fit and validation using full tumor growth time course for fitting. The whole-body mouse model was used to fit measurements of tumor xenograft volumes, and the tumor growth kinetic parameters were estimated. The predicted tumor volume over time is shown for the six datasets. **A,** Roland [34]. **B,** Zibara [35]. **C,** Tan [36]. **D,** Volk 2008 [37]. **E,** Volk 2011a [38]. **F,** Volk 2011b [38]. The model is able to reproduce experimental data for tumor growth without treatment and predict validation data not used in parameter fitting. Blue triangles and purple squares are control and treatment experimental data points, respectively. Shading indicates the 95% confidence interval. Note different scales on both axes.

We used nonlinear least squares optimization to fit the model and estimate the optimal parameter values, minimizing the error between the model predictions and the experimental measurements. Before pursuing model optimization, we first performed a global sensitivity analysis to identify which of the four tumor growth kinetic parameters most significantly influence the predicted tumor volume. We utilize the eFAST approach (described in the Methods), which we have routinely used in our previous work [28,39,40], to guide the model fitting. Results from the sensitivity analysis indicate that *k*_*0*_, *k*_*1*_, and *Ang*_*0*_ are influential parameters across all six data sets, where the total sensitivity index is greater than 0.4 (S1 Fig). Therefore, we estimated the values of these three tumor growth parameters, and we hold *ѱ* constant at a value of 20 [8]. We performed the model fitting 30 times for each of the six datasets (see Methods section for more details), obtaining 30 sets of optimized parameter values per dataset. Overall, the model does a good job of recreating the growth dynamics of untreated tumors (Fig 1, blue shading). One limitation is the fit to data from Volk *et al*. [38], where the model fails to capture the sigmoidal shape of the experimental tumor growth curve (Fig 1F).

We also explored an alternate equation for predicting tumor growth. In this case, we augment Eq (1) to include a coefficient (*C*_*Ang*_) that describes how dependent the tumor growth is on the concentration of the VEGF-VEGFR species (termed “Tumor Growth Model 2”):

$$\frac{dV(t)}{dt}=\frac{k_{0}*V(t)}{{\left[1+{\left(\frac{k_{0}}{k_{1}}*V(t)\right)}^{\psi }\right]}^{\frac{1}{\psi }}}∙\left(1-\frac{{Ang}_{0}-C_{Ang}∙Ang(t)}{{Ang}_{0}}\right)$$ (2)

We again applied the eFAST global sensitivity analysis to determine which of the five tumor growth kinetic parameters (*k*_*0*_, *k*_*1*_, *ѱ*, *Ang*_*0*_, and *C*_*Ang*_) significantly influence the predicted tumor volume. This analysis shows that the influence of *C*_*Ang*_ on the tumor volume is comparable to the effects of *k*_*0*_, *k*_*1*_, and *Ang*_*0*_. However, we find that the *C*_*Ang*_ parameter is tightly correlated to *Ang*_*0*_ (based on parameter identifiability analysis used in our previous work [40,41]). This means that it is not appropriate to fit both *C*_*Ang*_ and *Ang*_*0*_ at the same time, as their values may not be estimated with tight confidence intervals. Therefore, we moved forward with Tumor Growth Model 1, which includes four parameters that characterize the kinetics of tumor growth (Eq (1)), with three of the parameter values estimated in the model fitting described above. The estimated parameter values are listed in S3 Table.

### Model validation

We validated the model with data not used in the fitting. Using the same fitted kinetic parameters as the control case, we simulated the treatment regimens described in the *in vivo* mouse studies. The model does an excellent job of matching the experimental data (Fig 1, purple shading), capturing the effect of anti-VEGF treatment on tumor growth for the majority of datasets. Based on these results, the model is in agreement with experimental data of untreated tumor growth and can be appropriately validated using treatment data. Thus, our model is able to recreate the growth dynamics of untreated breast tumor xenografts in mice and can predict the tumor volume in response to anti-angiogenic treatment.

### Model fitting to early tumor growth data

**Fig 2:**
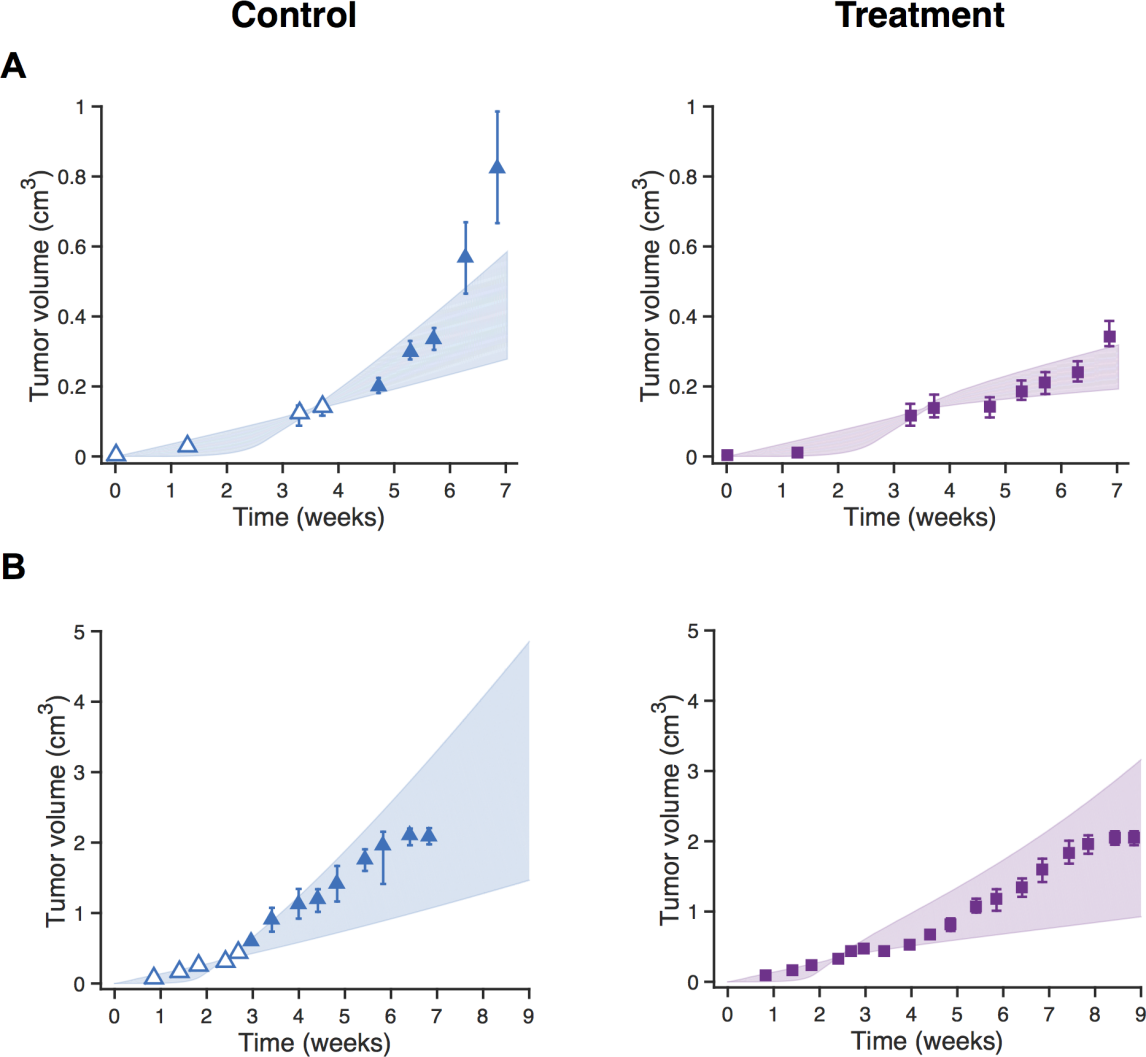
Model validation after fitting initial tumor growth data. Predicted tumor volume over time for the two datasets with at least three pre-treatment measurements for tumor volume. **A,** Roland [34]. **B,** Volk 2011a [38]. Experimental data points: triangles are control (left panel) and squares are treatment (right panel). Only the triangles outlined in blue are used for fitting. Shading shows the 95% confidence interval on the best fits. Note different scales on both axes.

We investigated whether it is possible to accurately predict the response to anti-VEGF treatment when the model fitting only includes the initial tumor growth data. We selected the datasets that included at least three tumor volume measurements prior to administration of bevacizumab (two out of the six datasets fit this criterion). We fit those initial experimental data points for the control (no anti-VEGF treatment) and validated the fitted model using the anti-VEGF treatment data. We again performed the model fitting 30 times for each dataset. The optimized model fit using only the initial tumor growth data was able to predict the tumor volume following treatment (Fig 2). Although the 95% intervals were wider in this fitting as compared to the results obtained when all of the data points were used for model fitting (see Fig 1), the newly optimized model still predicted reasonable values for the tumor size and the confidence intervals contained the experimental data points for validation (tumor volume with treatment), as shown in the right panels of Fig 2. These results demonstrate that the model can recreate treatment dynamics even when parameter fitting is performed using a limited number of experimental measurements. However, the estimated parameter values varied widely when fitting to the pre-treatment measurements only compared to fitting to all of the available control data (S3 Table). Therefore, we only used the model obtained by fitting the full set of control data to make meaningful comparisons amongst the parameter values from each dataset.

### Analysis of the estimated parameter values

**Fig 3:**
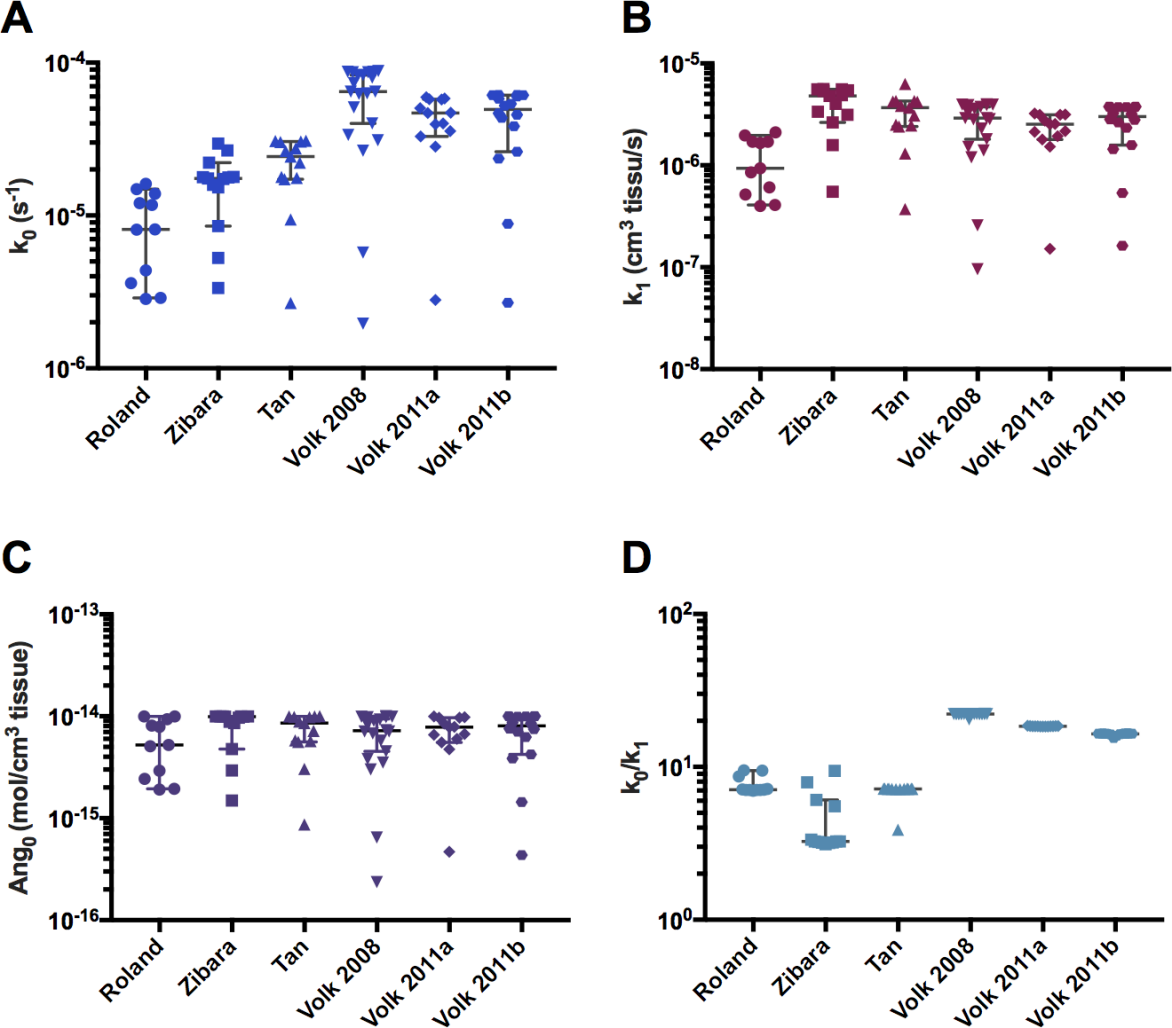
Estimated model parameters obtained from fitting. The whole-body mouse model was used to fit measurements of tumor xenograft volumes, and the tumor growth kinetic parameters were estimated. The estimated parameter values from the best fits are plotted for each dataset. **A,** *k*_*0*_. **B,** *k*_*1*_. **C,** *Ang*_*0*_. **D,** *k*_*0*_/*k*_*1*_. Horizontal bar indicates the median of the best fits obtained from fitting the model to each dataset; error bars indicate the 95% confidence interval. Statistical comparison of the estimated parameter sets is given in Fig 5.

We evaluated the optimized parameters estimated from fitting the model to all of the available control data. The estimated parameter values for the fits with the lowest errors are given in Fig 3. For all of the fitted parameters, the estimated 95% confidence intervals are within one order of magnitude or less, and there are few outliers. This indicates that the parameter values can be determined with high confidence, and allows statistical analysis to compare the parameter values obtained from fitting each dataset. Visual inspection shows that when fitting to the datasets from Volk et al., the model fitting and parameter estimation showed higher *k*_*0*_/*k*_*1*_ ratios than the other three datasets (Fig 3D). Since there appear to be other differences in the estimated parameter values, we wanted to determine if the differences in the parameter values influence the predicted response to anti-angiogenic treatment. Below, we present simulations obtained using the optimized parameter sets estimated from fitting the control data and compare the responses to treatment.

### Predicting the response to anti-VEGF treatment

**Fig 4:**
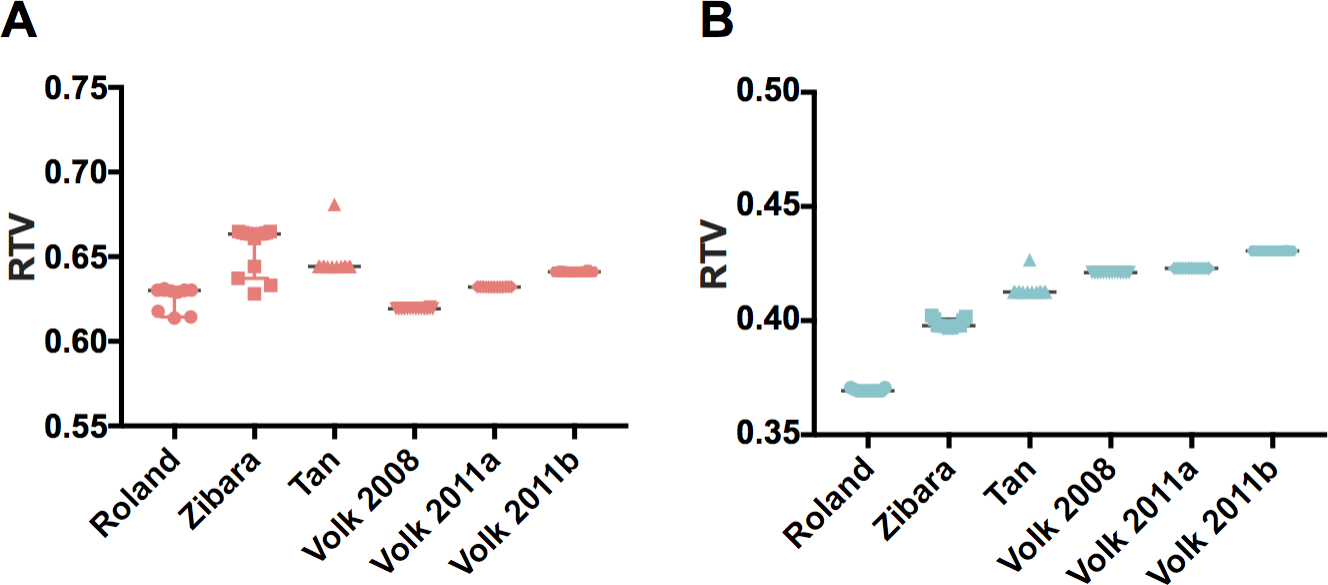
Predicted response to anti-VEGF treatment. The whole-body mouse model, including the dynamic tumor compartment whose volume is predicted using Eq (1), was used to simulate bevacizumab treatment at a dose of **A,** 2 mg/kg or **B,** 10 mg/kg. The relative tumor volume (RTV) predicted by the model is shown. Horizontal bar indicates the median of the predicted RTV for the best fits from each dataset.

Having validated our model, we used the optimized parameter sets to predict the tumor volume in response to anti-VEGF treatment. We ran the model for each of the six datasets, using all 30 sets of optimized parameter values. For each set of parameters, the model was simulated for three cases: no treatment (control) and two treatment conditions (2 and 10 mg/kg bevacizumab). For the treatment cases, twice-weekly injections were simulated, starting when the tumor volume reached 0.1 cm^3^ (termed “*T*_*start*_”). We selected this volume, since it is established that this is the critical time at which tumors typically start secreting higher levels of angiogenic factors in order to recruit the vasculature necessary to support further growth (~1–2 mm in diameter). For all cases, the model was simulated for 6 weeks after *T*_*start*_. We used the model to predict the relative tumor volume (RTV), the ratio of the final tumor volume for the control and treatment cases:

$$\mathrm{RTV}=\frac{V_{treatment}}{V_{control}}$$ (3)

where *V*_*treatment*_ and *V*_*control*_ are the tumor volumes at the end of the simulation with treatment and without treatment, respectively. Thus, the RTV represents the fold-change in tumor size due to treatment, where an RTV value less than one indicates that the treatment reduced the tumor volume, compared to the control. We use the RTV value to characterize the response to anti-VEGF treatment. The predicted responses to bevacizumab treatment at a dose of 2 or 10 mg/kg using the best fit parameter values are shown in Fig 4. The range of predicted RTV values indicates that certain tumors are more responsive to anti-VEGF treatment than others (Fig 4). In particular, the predicted RTV values obtained using fitted parameter values from fitting to data from Volk are higher than the predicted response for the other datasets for the 10 mg/kg dose. Interestingly, the ordering of the most responsive tumors differs for the two dosage levels, indicating nonlinear effects of the drug that vary with the amount administered. We next performed a thorough statistical comparison of the RTV and the estimated parameter values obtained in the fitting.

**Fig 5:**
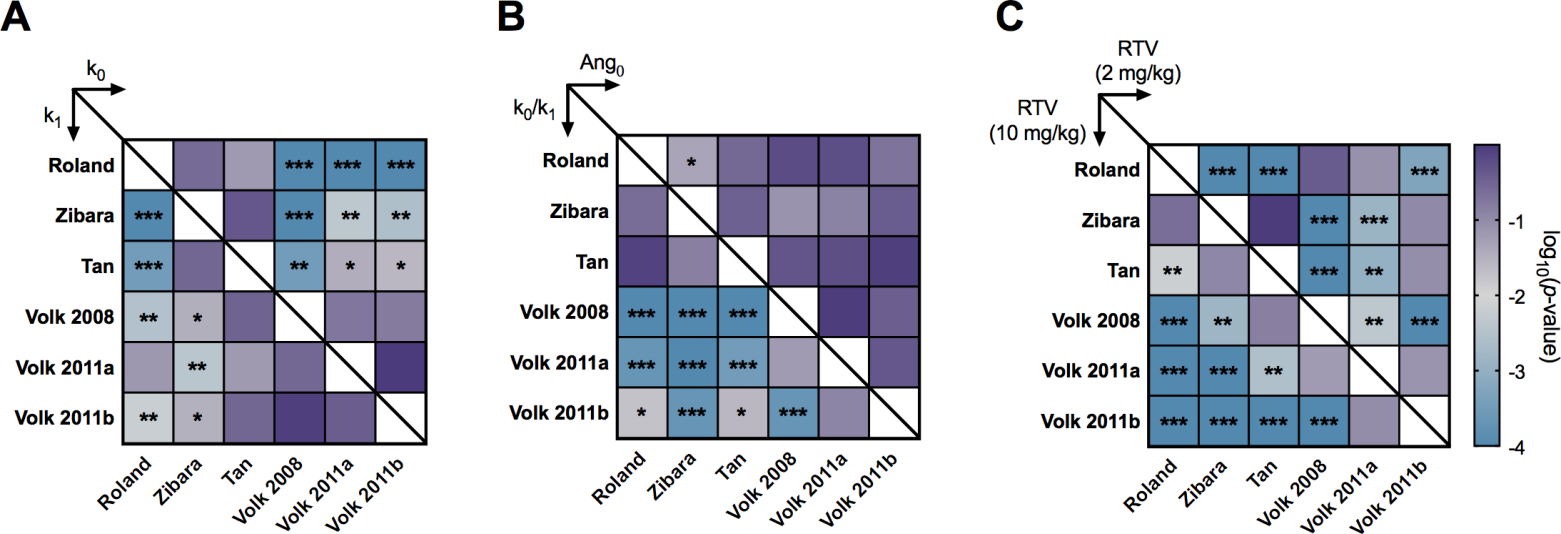
Statistical analysis of the optimized parameter sets. Standard ANOVA analysis followed by pairwise comparisons was used to determine whether the sets of optimized parameter values were statistically different. **A,** upper triangle: *k*_*0*_; lower triangle: *k*_*1*_. **B,** upper triangle: *Ang*_*0*_; lower triangle: *k*_*0*_/*k*_*1*_. **C,** upper triangle: RTV for bevacizumab dose of 2 mg/kg; lower triangle: RTV for dose of 10 mg/kg. The color and asterisks indicate log_10_(*p*-value): ***, (*p*-value ≤ 0.001); **, (0.001 < *p*-value ≤ 0.01); *, (0.01 < *p*-value < 0.05).

Our statistical analysis indicates a relationship between particular kinetic parameters that characterize tumor growth and the effectiveness of treatment. We used statistical analyses to determine whether the sets of estimated parameters or the predicted RTV values were statistically significantly different (*p* < 0.05) across the six datasets (Fig 5). Based on this analysis, we found that all datasets with significantly different predicted RTV values had significantly different *k*_*0*_, *k*_*1*_ or *k*_*0*_/*k*_*1*_ ratios. Interestingly, there was no statistically significant difference in the estimated *Ang*_*0*_ values, the “basal angiogenic signal”, between any of the datasets. Overall, the statistical analysis reveals that certain kinetic parameters (particularly, *k*_*0*_/*k*_*1*_) varied considerably between datasets and corresponded to significantly different treatment response (as indicated by the RTV value). The values of those parameters, which characterize the kinetics of tumor growth, may be used to predict the response to treatment.

### Determination of relationship between tumor growth parameters and response to treatment

**Fig 6:**
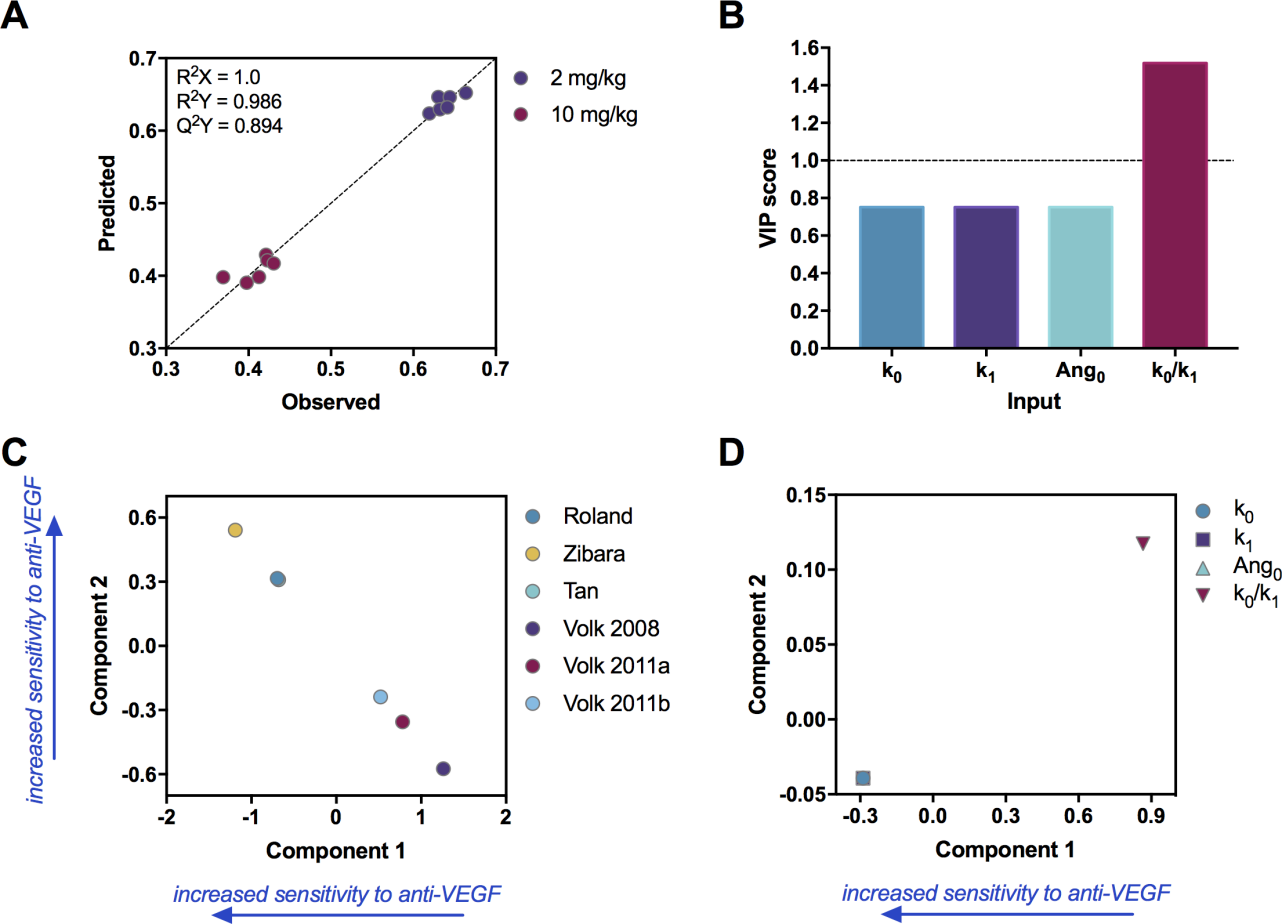
Results from multivariate analysis. PLSR analysis quantifies how the tumor growth parameters influence the response to treatment (RTV). **A,** PLSR model to predict RTV for two dosage levels of the anti-VEGF. The optimal PLSR model includes two components. Decreasing in component 1 or increasing in component 2 corresponds to higher efficacy of the anti-VEGF treatment. **B,** VIP scores for the model inputs; a score greater than one indicate variables that are important for predicting the RTV. **C,** Scores of the model output, revealing how tumors from each dataset compare in their responsiveness to treatment. **D**, Loadings of the model inputs, indicating how the model inputs (fitted parameters) correspond to sensitivity to anti-VEGF treatment.

We applied partial least squares (PLSR), a multivariate regression analysis, to further quantify the importance of specific tumor growth characteristics in predicting the response to anti-VEGF treatment. We used the values of *k*_*0*_, *k*_*1*_, *Ang*_*0*_, and *k*_*0*_/*k*_*1*_ as inputs (predictors) and the RTV at the two dosage levels for bevacizumab (2 and 10 mg/kg) as the responses. We determined the optimal PLSR model by varying the number of components from one to four and calculating the fitness metrics R^2^X, R^2^Y, and Q^2^Y values (see Methods section). We also varied the number of inputs, using different combinations of the estimated parameters. The fitness metrics for all PLSR models that we evaluated are listed in S4 Table. The final PLSR model (i.e., the model that best predicted the responses without over-fitting) had two components and included four inputs (*k*_*0*_, *k*_*1*_, *Ang*_*0*_, and *k*_*0*_/*k*_*1*_). This PLSR model is able to accurately predict the RTV at both dosage levels (Fig 6A), captures the variance in the inputs and outputs (high R^2^X and R^2^Y, respectively), and performs well with leave-one-out cross validation (Q^2^Y = 0.89). All PLSR models that included the *k*_*0*_/*k*_*1*_ ratio but excluded *k*_*0*_, *k*_*1*_, or *Ang*_*0*_ performed equally well in the cross validation analysis; however, the fitness metrics are the same, and we cannot objectively select one model over another. Therefore, we moved forward with the model that included all four inputs.

We analyzed the PLSR model to obtain insight regarding how the four inputs relate to the outputs. The variable importance of projection (VIP) scores for the four model inputs indicate that the value of *k*_*0*_/*k*_*1*_ is the largest contributor to predicting the RTV (Fig 6B). This suggests that the value of *k*_*0*_/*k*_*1*_ could be used to distinguish tumors that will respond to therapy or not.

Although the PLSR components do not explicitly correspond to a physiological variable, plotting the loadings for the inputs and outputs provides some insight into the meaning of each component. A plot of the loadings for the outputs (Fig 6C) reveals that both components capture the treatment efficacy. Here, we consider both components, as together, they account for 99% of the variance in the output. Decreasing in component 1 and increasing in component 2 corresponds to increased efficacy of the anti-VEGF treatment. The datasets in which anti-VEGF treatment is the least effective in reducing tumor growth (collectively, across the two drug doses) compared to the other datasets, have the highest loading in component 1 and lowest loading in component 2 (i.e., appearing in the lower right portion of the plot). In comparison, measurements from tumors in which anti-VEGF treatment leads to more growth inhibition appear in the upper left quadrant of the plot.

A plot of the loadings for the inputs reveals how the estimated tumor growth parameters are associated with treatment efficacy. We focus first on the loadings for component 1, as this component accounts for 94% of the variance in the inputs. We find that *k*_*0*_/*k*_*1*_ is positively correlated with low treatment efficacy, as it has a positive loading in component 1 (Fig 6D). The *k*_*0*_/*k*_*1*_ ratio also has the highest loading in component 2. Together, these results suggest that a high value of *k*_*0*_/*k*_*1*_ is associated with low treatment efficacy. In summary, the multivariate analysis provides a regression model that accurately predicts the relative tumor volume following anti-VEGF treatment, given the tumor growth parameters. Additionally, the analysis confirms the importance of *k*_*0*_/*k*_*1*_ as a key predictor of the tumor’s response to anti-VEGF treatment.

### Effect of tumor receptor number on the response to treatment

**Fig 7:**
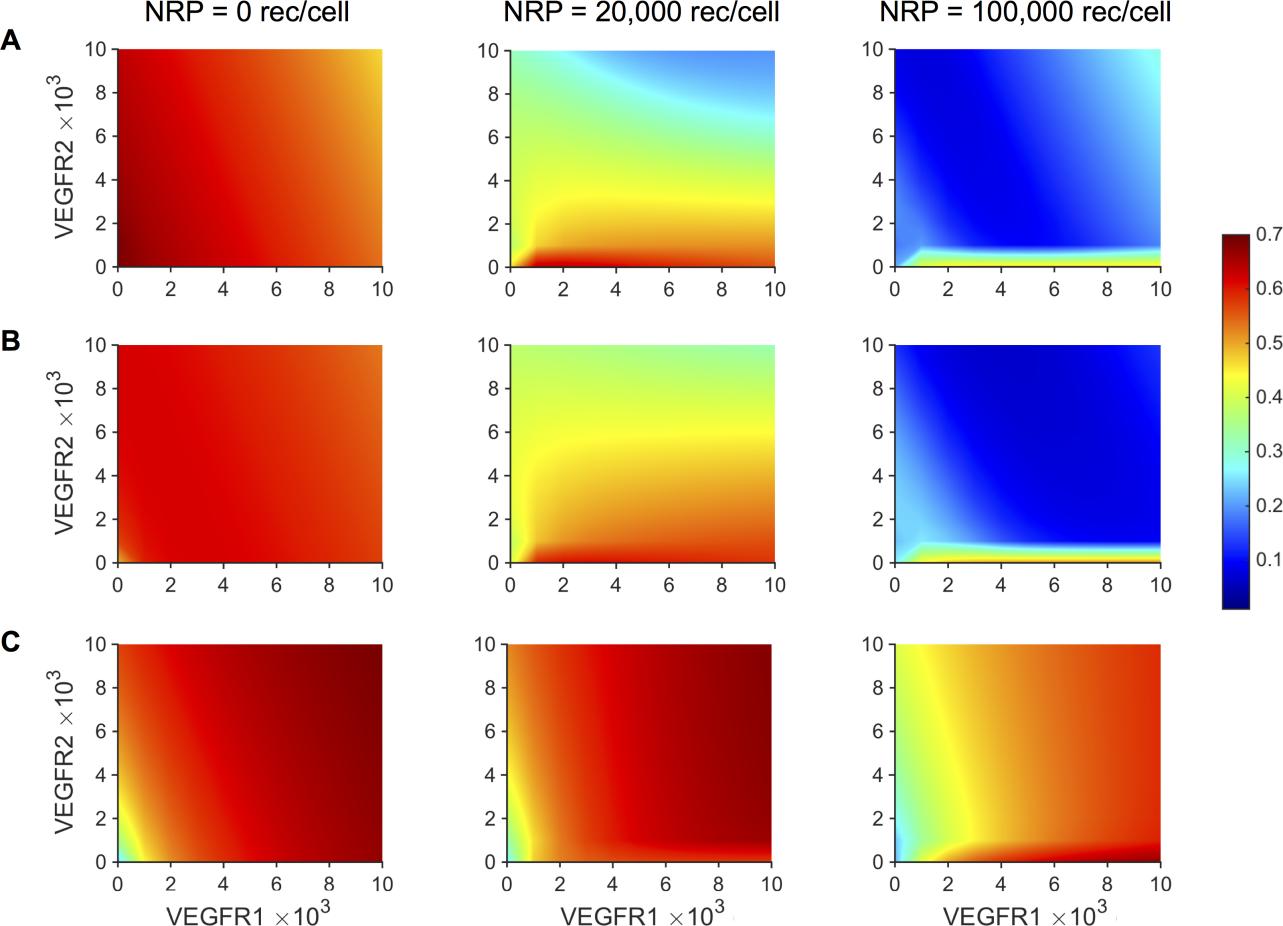
Effect of VEGF receptor expression on tumor cells. Relative tumor volume (RTV) predicted by the model using optimized parameter values obtained from fitting: **A,** Roland [34]. **B,** Zibara [35]. **C,** Volk 2008 [37], for different VEGF receptor levels on tumor cells. Neuropilin density varies: 0 receptors/cell (*left*), 20,000 receptors/cell (*center*), and 100,000 receptors/cell (*right*). Contour plots reveal the relationship between RTV and VEGFR1, VEGFR2, and neuropilin receptor density on tumor cells. The colorbar indicates the RTV value, with the same range for all panels. Red color indicates higher RTV, representing tumor conditions that are less favorable for anti-VEGF treatment.

After validating the model and investigating relationships between kinetic parameters describing tumor growth and response to treatment, we sought to investigate the effects of tumor-specific properties. In particular, we examined the effect of neuropilin and VEGF receptor levels on relative tumor volume. VEGF receptor levels were varied from 0 to 10,000 receptors/cell, and NRP levels were varied from 0 to 100,000 receptors/cell. Using a representative set of parameters from the best fits for each dataset, we used the model to determine *T*_*start*_ for each combination of receptor levels. We then ran the model to simulate the tumor growth for six weeks past *T*_*start*_ to obtain the baseline control volumes. Treatment volumes were obtained by simulating twice-weekly bevacizumab injections at a dose of 10 mg/kg for six weeks after *T*_*start*_. The RTV values were calculated for each combination of the tumor receptor densities. The model predicts that higher neuropilin levels led to increased treatment efficacy, especially for high VEGFR2 levels (Fig 7). The predicted RTV values obtained using the estimated parameters from certain datasets show that neuropilin expression has a noticeable impact on the response to treatment (Fig 7A and 7B). In comparison, neuropilin levels seem to have a diminished impact for the Volk dataset, indicated by contour plots that are very similar, even with drastic changes in neuropilin receptor levels (Fig 7C). In summary, the model can be used to study tumor-specific conditions that are favorable for anti-angiogenic treatment. Higher receptor expression is predicted to increase anti-VEGF efficacy, although the relationship was not uniform across all datasets, indicating the importance of accounting for specific tumor settings.

## Discussion

We have developed a compartmental model representing tumor-bearing mice in which the tumor volume is responsive to changes in VEGF concentration. The tumor volume explicitly depends on the “angiogenic signal”, which is the signal produced when VEGF binds to its receptors on tumor endothelial cells. In this way, the model can be applied to analyze the effect of anti-VEGF treatment on xenograft tumor growth, aiding in the analysis of pre-clinical data. Tumor growth kinetic parameters are obtained by fitting the model to experimental data of breast xenograft tumor growth in mice for control conditions (no anti-angiogenic treatment) and are validated with treatment data. By including a dynamic tumor volume that explicitly depends on the concentration of VEGF-bound receptors, we address a primary limitation of our previous work.

Our approach of training the model using control data and using the optimized model to predict treatment data is a significant advantage over previous modeling work. For example, in model fitting performed by other groups, tumor growth parameters were estimated by simultaneously fitting both control and treatment groups [33] or parameter values adopted from previous models [42]. In other work [8,33], the tumor growth equation includes coefficients that characterize the killing effect of cancer drugs, including anti-angiogenic agents, on tumor growth. In contrast, our computational model is able to accurately predict response to anti-VEGF treatment, validation data not used in the fitting. This is a significant feature of our model–it is trained using control data and can reproduce the response to anti-VEGF treatment simply by introducing the drug into the blood compartment, mimicking pre-clinical mouse studies.

The model provides unique insight into how certain kinetic parameters that characterize tumor growth correlate with response to anti-angiogenic treatment. Our results demonstrate how the parameters describing tumor growth could be used as a predictive biomarker for treatment response. In comparison, other studies have used volume-based growth tumor kinetics as a prognostic biomarker. Lee and coworkers found that the time to progression (defined as the time it takes the tumor to grow from its nadir in volume after treatment to a progressive disease state) was significantly correlated with overall survival [11]. In other work, researchers used tumor growth kinetics to determine the efficacy of anti-angiogenic treatment [12–15]. Excitingly, our approach is highly predictive, where volumetric measurements performed prior to treatment can give insight into how the tumor might respond to an anti-VEGF agent such as bevacizumab. This work is particularly useful in the pre-clinical setting–the model parameters can be systematically varied, and the tumor volume can be predicted for each case. Thus the model serves as a quantitative tool to perform *in silico* pre-clinical trials, guiding *in vivo* pre-clinical studies. It may be possible to extend the model to simulate human tumor growth in the future.

We performed various analyses to quantify how the tumor growth kinetic parameters influence the response to treatment. The PLSR and statistical analyses reveal that higher *k*_*0*_*/k*_*1*_ values are related to decreased treatment efficacy. In nearly all cases of the pairwise comparisons, datasets with significantly different responses to anti-VEGF treatment, the *k*_*0*_/*k*_*1*_ ratio is also significantly different. Our statistical analyses indicate a direct relationship between the *k*_*0*_/*k*_*1*_ ratio and effectiveness of treatment. Simeoni *et al*. posit that *k*_*0*_ and *k*_*1*_ may be indicative of the initial aggressiveness of the cell line and of the response of the animal to tumor progression (i.e., immunological or anti-angiogenic response), respectively [33]. According to this interpretation, treatment would be least effective for tumors with aggressive initial growth (high *k*_*0*_) combined with a strong response from the animal (low *k*_*1*_). Additionally, we find that the basal angiogenic signal, *Ang*_*0*_, is not predictive of anti-angiogenic treatment response. This agrees with experimental results indicating that the ability of basal levels of circulating angiogenic factors to predict treatment efficacy is limited [7].

We used the model to investigate how the number of VEGF receptors and co-receptors on tumor cells influences the response to treatment. Currently, modified expression of VEGF receptors (VEGFR1, VEGFR2, or NRP1) appears to be among the most promising markers for bevacizumab treatment, though this has not consistently been replicated across different studies involving various cancer types [5]. In particular, low levels of soluble VEGFR1 expression in plasma and NRP1 expression on tumor cells are characteristics of a bevacizumab-responsive tumor [5]. Therefore, we wanted to use our model to predict the influence of tumor-specific properties on treatment efficacy. The model predicts that low levels of VEGFR lead to increased treatment efficacy for all datasets. The treatment is predicted to be most effective when tumor NRP levels are high. These results are in agreement with other biomarker studies [9,43]. Although there was a consistent relationship between receptor levels and treatment efficacy, the extent to which receptor numbers influenced the predicted relative tumor volume was not identical for all tumors. Datasets for tumors with higher *k*_*0*_/*k*_*1*_ ratios had higher RTV (i.e., the treatment was less effective), even for a wide range of receptor expression levels. This may indicate that intrinsic characteristics of the tumor related to its growth kinetics make anti-angiogenic treatment less effective, regardless of microenvironmental tumor conditions. As a result, solely using receptor expression as a predictive biomarker could lead to inconsistent results across tumor types.

The focus of our model is on the molecular level interactions occurring between VEGF and its receptors. In our model, the number of VEGF-receptor (pro-angiogenic) signaling complexes formed directly influences tumor growth. We acknowledge that this representation of tumor growth omits the intracellular signaling pathways and corresponding cellular-level responses (i.e., proliferation and migration) involved in new blood vessel formation. However, the model does indeed capture the dynamics of tumor growth, providing a mechanistic understanding of the growth kinetics that contribute to the response to anti-VEGF treatment.

We acknowledge some assumptions and limitations that may be addressed as more quantitative data become available. We do not account for changes in tumor vascularity relative to the tumor volume. The tumor volume consists of interstitial space, vascular volume, and tumor cells. We account for tumor growth by assuming the tumor cell volume fraction increases, while the interstitial space volume fraction decreases, and the relative proportion of the vascular volume is constant (see Methods section for more detail). This means that the tumor vascularity does change as the overall tumor volume grows, but it remains in the same proportion relative to the whole tumor volume. Furthermore, we do not simulate remodeling of the blood compartment or changes in vascular permeability in response to anti-VEGF treatment. However, experimental data show a decrease in microvessel density following bevacizumab treatment [44], and incorporating this observation would enhance the model. Additionally, anti-angiogenic treatment promotes normalization of the vasculature, which allows for more efficient delivery of chemotherapy to the tumor [45]. Accounting for changes in the microvascular density would allow us to simulate combination treatments that include chemotherapy and anti-angiogenic agents. Unfortunately, there is a lack of robust time-series data that can be used to predict changes in vascular density with treatment. This limitation may be addressed as additional quantitative measurements are published.

The model is highly successful in capturing the growth kinetics of exponential or linear growth curves. However, the model does not accurately predict sigmoidal tumor growth. The equation governing tumor growth used in our model is based on the foundational work of Simeoni *et al*., who adapted a Gompertz model of tumor growth to investigate both the exponential and linear phases of growth [33]. Although this makes the tumor growth equation more flexible, it also limits the ability to simulate an eventual plateau in growth. The model’s inability to capture sigmoidal growth was particularly apparent when fitting the Volk datasets [37,38]. However, we have focused on exponential growth, as it has been implemented in many other mathematical models [46,47] and shown to accurately fit tumor growth data [48]. Expansion of the tumor growth equation can be added in future studies.

### Concluding thoughts

We constructed a computational model that simulates the kinetics of VEGF binding to its receptors and the influence of VEGF-bound receptor complexes on tumor volume in tumor-bearing mice. The validated model accurately predicts the tumor growth upon administration of anti-angiogenic treatment that targets VEGF. The fitted parameter values estimated in the present study point to the possibility of using tumor growth kinetics as a predictive biomarker for anti-angiogenic treatment. Additionally, this model also helps to elucidate why biomarker candidates such as expression of VEGF receptors on tumor cells may not be reliable for all tumors. Although the model predicts that receptor levels influence response to treatment, the effects are not uniform across all of the experimental datasets we analyzed. Thus, our modeling work lays the foundation for future studies to investigate the importance of tumor growth kinetics as a predictive and specific biomarker and can accelerate the discovery of biomarker candidates in pre-clinical studies.

## Materials and methods

### Computational modeling

#### Compartmental model

**Fig 8:**
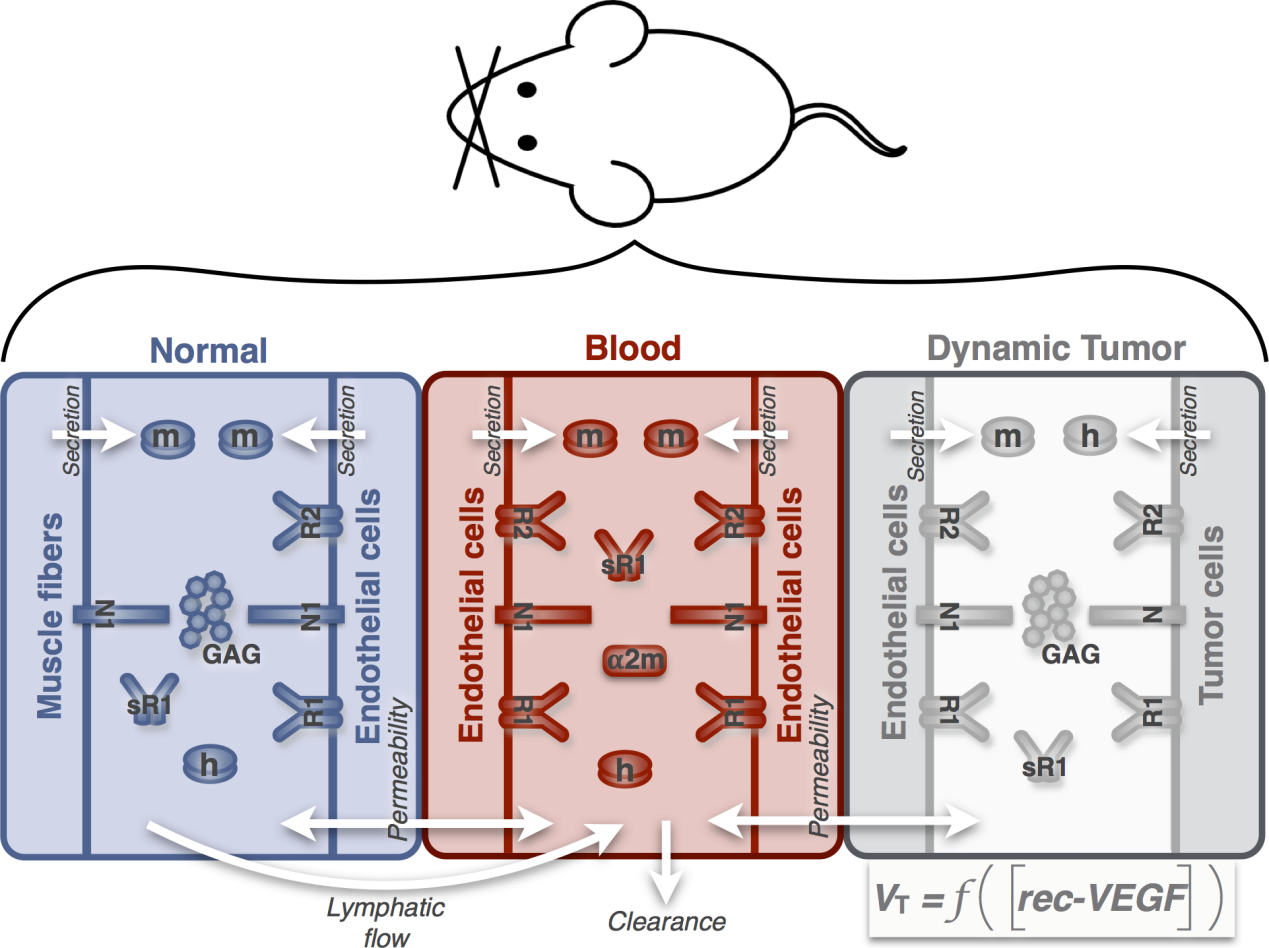
Model schematic. The computational model includes three compartments: normal tissue, blood, and tumor volume. The compartments are connected via lymphatic flow from the interstitial space in the normal tissue to the blood and transendothelial macromolecular permeability. Molecular species include human and mouse VEGF isoforms, VEGF receptors and co-receptors (including the soluble receptor VEGFR1, sR1), and the protease inhibitor α-2-macroglobulin (a2m). Glycosaminoglycan (GAG) chains represent the extracellular matrix. The volume of the tumor depends on the concentration of receptor-bound VEGF complexes on tumor endothelial cells (denoted as [rec-VEGF]).

In this work, we expand on our previous three-compartment model [27] by including VEGF-mediated tumor growth. We briefly describe the full model and detail the new additions that are the focus of this work. The model is comprised of three compartments representing the whole mouse: normal tissue (assumed to be skeletal muscle), blood, and tumor (Fig 8). The model includes human and mouse VEGF isoforms: human isoforms (VEGF_121_ and VEGF_165_) are secreted by tumor cells, and mouse isoforms (VEGF_120_ and VEGF_164_) are secreted by endothelial cells in the normal, blood, and tumor compartments and muscle fibers in the normal tissue. VEGF receptors (VEGFR1 and VEGFR2) and co-receptors (neuropilins) are expressed on the surface of muscle fibers, endothelial cells, and tumor cells. VEGFR1 and VEGFR2 are the primary receptors to which VEGF binds. The neuropilins (NRP1 and NRP2) are co-receptors for VEGF, to which VEGF can directly bind. Additionally, NRPs can couple to VEGF receptors VEGFR1 or VEGFR2, and then VEGF can bind to the VEGFR-NRP complex. The interactions between VEGF and its receptors and co-receptors occur in all three compartments. By binding to its receptors on endothelial cells in the tumor compartment, VEGF is able to initiate pro-angiogenic signaling that mediates the formation of new blood vessels. We account for VEGF-mediated tumor growth by incorporating the concentration of VEGF-bound receptors into the tumor volume equation (described in more detail below). Parameters characterizing the compartment geometry, receptor densities, kinetic rates, and transport rates are given in S1 Dataset [27].

#### Tumor volume and growth

Previously, we assumed the tumor volume increased exponentially with time, based on measurements from tumor xenografts [27]. Under that assumption, cancer treatment, including anti-angiogenic therapy, has no effect on tumor growth. In the present study, we address that limitation by introducing an equation for tumor growth wherein the volume of the tumor compartment is dependent on the “angiogenic signal” (*Ang*) produced when VEGF binds to its receptors on endothelial cells in the tumor. By including the concentration of VEGF-bound receptors directly into the tumor volume equation, we account for VEGF-mediated tumor angiogenesis and subsequent tumor growth.

The tumor compartment is assumed to consist of cancer cells, endothelial cells (vascular volume) and interstitial space, each of which has a defined volume fraction (i.e., volume relative to the total tumor volume). Our previous model assumed that as the total tumor volume increased, the relative proportions of cancer cells, vascular space, and interstitial space remain constant. Here, we still have the volume fraction for the vascular space remaining constant, based on a range of experimental data [49–51]. However, we used results from a recent imaging study to account for an increase in the relative volume of cancer cells as the tumor volume increases. Christensen and coworkers measure how tumor cell density increases as the tumor grows by tracking cancer cells in xenograft tumors in rats using near near-infrared (NIR) fluorescence dyes [52]. The authors quantify the fluorescence intensity in a tumor and use it to estimate the number of cancer cells as the tumor grows over time. The estimated cell count was normalized by the tumor volume to obtain the number of cancer cells per unit volume of tumor tissue as the tumor grows. We extracted the values obtained by Christensen and coworkers for MDA-MB-231 tumors and converted them to the cancer cell volume fraction using the volume of tumor cells, as we have done in our previous work [27]. Therefore, we have been able to incorporate into our model an increase in the cancer cell volume fraction over time. Assuming a tumor cell volume of 905 μm^3^, based on our previous examination of the literature [27], we developed expressions describing the decay of interstitial space during tumor growth. We found that the relative decrease in interstitial space during tumor growth was adequately modeled by exponential decay. The equations for how the relative volume of the interstitial space varies with total tumor volume are given in S5 Table.

### Data extraction

Data from six independent *in vivo* published experimental studies of MDA-MB-231 xenograft tumors in mice were used for parameter fitting and validation [34–38]. The six datasets included growth profiles for untreated tumors (control), as well as tumors treated with the anti-VEGF agent bevacizumab. Experimental data was extracted using the WebPlotDigitizer program (http://arohatgi.info/WebPlotDigitizer). The numerical values are provided in S1 Table.

The extended Fourier amplitude sensitivity test (eFAST), a global variance-based sensitivity analysis, was used to understand how different parameters (“model inputs”) affect model predictions (“model outputs”). In this method, the inputs are varied together within specific ranges at different frequencies, and the model outputs are calculated. The Fourier transform of a model output is then calculated to identify which inputs have the most influence based on the amplitude of each input’s frequency, where greater amplitudes indicate more sensitive parameters. By varying the inputs at the same time, this method allows for the calculation of the total FAST index, *S*_*Ti*_, for each input *i*. The total index is a measure of the global sensitivity, accounting for second and higher-order interactions between multiple inputs.

We implemented the eFAST method using MATLAB code developed by Kirschner and colleagues. We analyzed the effects of the tumor growth parameters (*k*_*0*_, k_1_, *ѱ*, and *Ang*_*0*_) on one model output (the tumor volume without anti-VEGF treatment). The parameter values were allowed to vary at least one order of magnitude (10^−8^ to 10^−2^ for *k*_*0*_ and *k*_*1*_, 0.1 to 50 for *ѱ*, and 10^−16^ to 10^−14^ for *Ang*_*0*_) to account for potentially large uncertainty in the model parameters. These are the same ranges used for the parameter estimation (described below). The parameters for which the total FAST index is large are considered to be influential parameters, and their values are estimated in the model fitting.

### Parameter estimation

#### Model training

We fit the influential tumor growth parameters (“free parameters”) using the control tumor growth profiles for each dataset. Each of the six datasets provides measurements of the tumor volume in the mouse xenograft in vivo model, where MDA-MB-231 cells were injected into mice. However, there are significant differences between the six studies, as outlined in S2 Table. These include differences in the mouse strains used, the number of cancer cells injected to initiate tumor growth, whether the cancer cells were injected alone or with matrigel, and the site of the cancer cell injection. Additionally, the equation used to calculate the tumor volume influences the reported volume, and papers use different volume equations. Given all of these differences, we treat each dataset individually. This is analogous to determining patient-specific tumor growth parameters, even for patients with the same type of tumor.

Fitting was performed using the *lsqnonlin* function in MATLAB to minimize the sum of squared residuals (SSR):

$$\min\;SSR(\Theta )=min\sum_{i=1}^{n}{\left(V_{exp,i}-V_{sim,i}(\Theta )\right)}^{2}$$ (4)

where *V*_*exp*,*i*_ is the *i*th experimentally measured tumor volume, *V*_*sim*,*i*_ is the *i*th simulated volume at the corresponding time point, and *n* is the total number of experimental measurements. The minimization is subject to Θ, the set of upper and lower bounds on each of the free parameters. We found that weighting the residual by the experimental measurement biased the error towards early data points and reduced the model’s ability to fit the full course of tumor growth. Therefore, we minimized the residual, with no weighting, to fit the model to the experimental data.

We performed the parameter fitting 30 times for each dataset. To attempt to arrive at a global minimum for the error, we initialized each fitting run by randomly selecting a value for the free parameters within the specified upper and lower bounds. The bounds were set such that the range for each parameter was at least one order of magnitude: 10^−8^ to 10^−2^ for *k*_*0*_ and *k*_*1*_ and 10^−16^ to 10^−14^ for *Ang*_*0*_. After performing the model fitting, we used the SSR to identify the optimal parameters. Parameter sets with the smallest errors were taken to be the “best” fits and were used for subsequent statistical analysis. The number of “best” parameter sets varied between datasets and ranged from 11 to 20 parameter sets. We first tested to see whether there were significant effects of the experimental data being fit on the estimated parameters values using one-way non-parametric ANOVA. This method makes no assumptions about the distributions of parameter values and tests whether samples originate from a common distribution. We then performed post-hoc analyses to make pairwise comparisons using the Kruskal-Wallis test. We corrected for multiple comparisons by controlling the false discovery rate. All statistical analyses were performed using GraphPad Prism.

Two of the experimental datasets contained at least three data points prior to administration of treatment [34,38]. These points were used in a separate model fitting to see whether limiting the data used for model training to only pre-treatment measurements could generate a fitted model that still accurately predicts the response to anti-angiogenic treatment.

#### Model validation–anti-VEGF drug treatment

After fitting the control data, we validated the estimated parameters with data not used in the fitting. We applied the fitted model to simulate anti-angiogenic treatment (bevacizumab, a monoclonal antibody that binds to the human VEGF isoforms) and compared the predicted tumor growth profile to the experimental measurements for the treatment cases. Here, we simulated the dosing regimens used in each experiment with the same optimized parameters obtained by fitting the control data. For each dataset, we simulated intravenous injections lasting for one minute (as in our previous model). More specifically, this means that there is a net rate of secretion of the drug directly into the blood compartment. All six experimental studies administered bevacizumab twice-weekly; however, the dosage varied between the studies. The dosing regimens are given in S2 Table. The binding affinity and clearance rate for bevacizumab were obtained from experimental studies in which VEGF was immobilized on a flow cell (FC) and bevacizumab was injected over the FC at varying concentrations [53]. Based on those measurements, the binding affinity was set to 4456 pM (*k*_*on*_ = 5.4×10^4^ M^-1^s^-1^; *k*_*off*_ = 2.19×10^−5^ s^-1^), and 5.73×10^−7^ s^-1^ was used for the anti-VEGF clearance rate.

### Partial least squares regression analysis

Partial least squares regression (PLSR) modeling was used to determine the relationship between the fitted parameters characterizing tumor growth kinetics (inputs) and the response to treatment given by the RTV value (output). PLSR modeling seeks to maximize the correlation between the inputs and outputs. To accomplish this, the inputs and outputs are recast onto new dimensions called principal components (PCs), which are linear combinations of the inputs. The regression coefficients estimated by PLSR describe the relative importance of each input. Quantitative measures from the PLSR modeling, including the loadings and scores, provide some insight into the biological meaning of the PCs [54]. Additionally, we use the estimated regression coefficients to determine each input’s contribution across all responses. This metric is given by the “variable importance of projection” (VIP) for each predictor. The VIP value is the weighted sum of each input’s contribution to all of the responses. As such, the VIP values indicate the overall importance of the predictors. VIP values greater than one indicate variables that are important for predicting the output response.

In the final PLSR model we selected, the input matrix was 6 rows x 4 columns, where the 6 rows correspond to the best fit for each of the six datasets, and the 4 columns consisted of the estimated free parameters (*k*_*0*_, *k*_*1*_, and *Ang*_*0*_) and the calculated ratio of k_0_/k_1_. The output matrix was 6 rows x 2 columns, where the rows corresponds to the predicted RTV using the best fit for each of the six datasets, and the columns are the two treatment doses (2 and 10 mg/kg). We used two metrics to evaluate the model fitness: R^2^Y and Q^2^Y, both of which have a maximum value of 1. The R^2^Y value indicates how well the model fits the output data. The Q^2^Y metric specifies how much of the variation in the output data the model predicts [55], and values greater than 0.5 indicate that the model can predict data not used in the fitting. We performed PLSR modeling using the nonlinear iterative partial least squares (NIPALS) algorithm [56], implemented in MATLAB (Mathworks, Inc.). We implemented many other PLSR models, using various combinations of the four model inputs. The fitness metrics for each model evaluated are given in S4 Table.

### Numerical implementation

All model equations were implemented in MATLAB using the SimBiology toolbox. The final model is provided as the SimBiology project file, as SBML, and as a MATLAB m-file (S2 Dataset). Parameter fitting was performed using the *lsqnonlin* function MATLAB. GraphPad Prism was used to run statistical analyses on parameter values.

## Supporting information

S1 Fig Sensitivity indices of tumor growth parameters. The sensitivity indices estimated using the extended Fourier Amplitude Sensitivity Test (eFAST) quantifying the variance in the model output (tumor volume without treatment) with respect with covariances in combinations of model inputs: the tumor growth parameters *k*_*0*_, *k*_*1*_, *ѱ*, and *Ang*_*0*_ at distinct times for each dataset. **A,** Roland [34]. **B,** Zibara [35]. **C,** Tan [36]. **D,** Volk 2008 [37]. **E,** Volk 2011a [38]. **F,** Volk 2011b [38]. The sensitivity indices for the growth parameters are compared to a dummy variable that is not included in the model. Indices that are significantly different from the dummy variable influence the model output. We used a cutoff of 0.4 to select which parameters to fit in the parameter estimation.
(PDF)

S1 Table Experimental data extracted from published papers. Experimental data used for model fitting.
(XLSX)

S2 Table Experimental treatment from published papers. This table lists details regarding the experimental conditions of the studies used for model fitting.
(XLSX)

S3 Table Estimated parameter sets. This table lists the estimated parameter values for *k*_*0*_, *k*_*1*_, and *Ang*_*0*_ obtained from fitting to all of the control volume measurements (Roland [34], Zibara [35], Tan [36], Volk 2008 [37], Volk 2011a [38], and Volk 2011b [38]) and for fitting to the datasets with at least three tumor volume measurements before anti-angiogenic treatment began (Roland [34] and Volk 2011a [38]). The calculated errors for the training and validation data are shown, along with the calculated *k*_*0*_/*k*_*1*_ ratio.
(XLSX)

S4 Table Fitness metrics for PLSR models. We evaluated several PLSR models to evaluate the ability of the estimated tumor growth parameters (*k*_*0*_, *k*_*1*_, *Ang*_*0*_, and *k*_*0*_/*k*_*1*_) to predict the response to treatment (RTV). We examined the predictive ability of PLSR models that included all parameters, excluded one at a time, or excluded two at a time. We report the fitness metrics (R^2^X, R^2^Y, and Q^2^Y) and the VIP scores for the inputs included in the model.
(XLSX)

S5 Table Equations describing change in relative volume of the interstitial space. This table presents the equations for how the relative volume of the interstitial space changes as a function of the total tumor volume. This equation is unique for each of the datasets investigated.
(PDF)

S1 Dataset Detailed description of computational model. This file contains a description of the three-compartment computational model, including parameter values and initial conditions.
(PDF)

S2 Dataset Model file. This zipped file contains the computational model in multiple formats: the MATLAB SimBiology project file (.sbproj), SBML model (.xml) and MATLAB *m*-file (.m). To facilitate reproducibility, we provide a driver file that runs the *m*-file and simulates the control and treatment conditions (2 mg/kg) for a representative parameter set estimated from fitting to the Roland dataset.
(ZIP)

## References

[1] Olsson A, Dimberg A, Kreuger J, Claesson-Welsh L. VEGF receptor signalling—in control of vascular function. Nat Rev Mol Cell Biol. 2006;7: 359–371. doi: 10.1038/nrm1911 16633338 10.1038/nrm1911

[2] Carmeliet P, Jain RK. Molecular Mechanisms and and clinical applications of angiogenesis. Nature. 2011;473: 298–307. doi: 10.1038/nature10144 21593862 10.1038/nature10144 PMC4049445

[3] Al-Husein B, Abdalla M, Trepte M, DeRemer DL, Somanath PR. Antiangiogenic therapy for cancer: An update. Pharmacotherapy. 2012 pp. 1095–1111. doi: 10.1002/phar.1147 23208836 10.1002/phar.1147 PMC3555403

[4] FDA Approval for Bevacizumab—National Cancer Institute [Internet]. [cited 24 Apr 2017]. Available: https://www.cancer.gov/about-cancer/treatment/drugs/fda-bevacizumab

[5] Lambrechts D, Lenz HJ, De Haas S, Carmeliet P, Scherer SJ. Markers of response for the antiangiogenic agent bevacizumab. J Clin Oncol. 2013;31: 1219–1230. doi: 10.1200/JCO.2012.46.2762 23401453 10.1200/JCO.2012.46.2762

[6] Alberto MJ, Escobar M, Lopes G, Glück S, Vogel C. Bevacizumab in the Treatment of Metastatic Breast Cancer: Friend or Foe? Curr Oncol Rep. 2012;14: 1–11. doi: 10.1007/s11912-011-0202-z 22012632 10.1007/s11912-011-0202-z PMC3266439

[7] Jain RK, Duda DG, Willett CG, Sahani D V, Zhu AX, Loeffler JS, et al Biomarkers of response and resistance to antiangiogenic therapy. Nat Rev Clin Oncol. 2009;6: 327–338. doi: 10.1038/nrclinonc.2009.63 19483739 10.1038/nrclinonc.2009.63 PMC3057433

[8] Sharan S, Woo S. Quantitative Insight in Utilizing Circulating Angiogenic Factors as Biomarkers for Antiangiogenic Therapy: Systems Pharmacology Approach. CPT Pharmacometrics Syst Pharmacol. 2014;3: e139 doi: 10.1038/psp.2014.36 25295574 10.1038/psp.2014.36 PMC4474166

[9] Van Cutsem E, de Haas S, Kang Y-K, Ohtsu a., Tebbutt NC, Ming Xu J, et al Bevacizumab in Combination With Chemotherapy As First-Line Therapy in Advanced Gastric Cancer: A Biomarker Evaluation From the AVAGAST Randomized Phase III Trial. J Clin Oncol. 2012;30: 2119–2127. doi: 10.1200/JCO.2011.39.9824 22565005 10.1200/JCO.2011.39.9824

[10] Kopetz S, Hoff PM, Morris JS, Wolff RA, Eng C, Glover KY, et al Phase II trial of infusional fluorouracil, irinotecan, and bevacizumab for metastatic colorectal cancer: Efficacy and circulating angiogenic biomarkers associated with therapeutic resistance. J Clin Oncol. 2010;28: 453–459. doi: 10.1200/JCO.2009.24.8252 20008624 10.1200/JCO.2009.24.8252 PMC2815707

[11] Lee JH, Lee HY, Ahn M-J, Park K, Ahn JS, Sun J-M, et al Volume-based growth tumor kinetics as a prognostic biomarker for patients with EGFR mutant lung adenocarcinoma undergoing EGFR tyrosine kinase inhibitor therapy: a case control study. Cancer Imaging. Cancer Imaging; 2016;16: 5 doi: 10.1186/s40644-016-0063-7 26984681 10.1186/s40644-016-0063-7 PMC4794857

[12] Seyal AR, Parekh K, Arslanoglu A, Gonzalez-Guindalini FD, Tochetto SM, Velichko YS, et al Performance of tumor growth kinetics as an imaging biomarker for response assessment in colorectal liver metastases: correlation with FDG PET. Abdom Imaging. Springer US; 2015;40: 3043–3051. doi: 10.1007/s00261-015-0546-1 26353898 10.1007/s00261-015-0546-1

[13] El Sharouni SY, Kal HB, Battermann JJ. Accelerated regrowth of non-small-cell lung tumours after induction chemotherapy. Br J Cancer. 2003;89: 2184–9. doi: 10.1038/sj.bjc.6601418 14676792 10.1038/sj.bjc.6601418 PMC2395273

[14] Stein WD, Yang J, Bates SE, Fojo T. Bevacizumab reduces the growth rate constants of renal carcinomas: a novel algorithm suggests early discontinuation of bevacizumab resulted in a lack of survival advantage. Oncologist. 2008;13: 1055–1062. doi: 10.1634/theoncologist.2008-0016 18827177 10.1634/theoncologist.2008-0016 PMC3306833

[15] Rezai P, Yaghmai V, Tochetto SM, Galizia MS, Miller FH, Mulcahy MF, et al Change in the growth rate of localized pancreatic adenocarcinoma in response to gemcitabine, bevacizumab, and radiation therapy on MDCT. Int J Radiat Oncol Biol Phys. 2011;81: 452–459. doi: 10.1016/j.ijrobp.2010.05.060 21570199 10.1016/j.ijrobp.2010.05.060

[16] Day C-P, Merlino G, Van Dyke T. Preclinical Mouse Cancer Models: A Maze of Opportunities and Challenges. Cell. Elsevier; 2017;163: 39–53. doi: 10.1016/j.cell.2015.08.068 26406370 10.1016/j.cell.2015.08.068 PMC4583714

[17] Le Magnen C, Dutta A, Abate-Shen C. Optimizing mouse models for precision cancer prevention. Nat Rev Cancer. Nature Publishing Group, a division of Macmillan Publishers Limited. All Rights Reserved.; 2016;16: 187–196. doi: 10.1038/nrc.2016.1 26893066 10.1038/nrc.2016.1 PMC5517771

[18] Manning HC, Buck JR, Cook RS. Mouse Models of Breast Cancer: Platforms for Discovering Precision Imaging Diagnostics and Future Cancer Medicine. J Nucl Med. 2016;57: 60S–68S. doi: 10.2967/jnumed.115.157917 26834104 10.2967/jnumed.115.157917 PMC6231414

[19] Denayer T, Stöhr T, Van Roy M. Animal models in translational medicine: Validation and prediction. New Horizons Transl Med. 2014;2: 5–11. doi: http://dx.doi.org/10.1016/j.nhtm.2014.08.001

[20] Altrock PM, Liu LL, Michor F. The mathematics of cancer: integrating quantitative models. Nat Rev Cancer. Nature Publishing Group, a division of Macmillan Publishers Limited. All Rights Reserved.; 2015;15: 730–745. doi: 10.1038/nrc4029 26597528 10.1038/nrc4029

[21] Masoudi-Nejad A, Wang E. Cancer modeling and network biology: Accelerating toward personalized medicine. Semin Cancer Biol. 2015;30: 1–3. doi: 10.1016/j.semcancer.2014.06.005 24969134 10.1016/j.semcancer.2014.06.005

[22] Yankeelov TE, An G, Saut O, Luebeck EG, Popel AS, Ribba B, et al Multi-scale Modeling in Clinical Oncology: Opportunities and Barriers to Success. Ann Biomed Eng. 2016;44: 2626–2641. doi: 10.1007/s10439-016-1691-6 27384942 10.1007/s10439-016-1691-6 PMC4983505

[23] Kholodenko N. B, Yaffe MB, Kolch W. Computational approaches for analyzing information flow in biological networks. Sci Signal. 2012;5: re1 doi: 10.1126/scisignal.2002961 22510471 10.1126/scisignal.2002961

[24] Jain RK. Normalizing tumor microenvironment to treat cancer: Bench to bedside to biomarkers. J Clin Oncol. 2013;31: 2205–2218. doi: 10.1200/JCO.2012.46.3653 23669226 10.1200/JCO.2012.46.3653 PMC3731977

[25] Jubb AM, Miller KD, Rugo HS, Harris AL, Chen D, Reimann JD, et al Impact of exploratory biomarkers on the treatment effect of bevacizumab in metastatic breast cancer. Clin Cancer Res. 2011;17: 372–381. doi: 10.1158/1078-0432.CCR-10-1791 21224365 10.1158/1078-0432.CCR-10-1791 PMC3023787

[26] Wehland M, Bauer J, Magnussion NE, Infanger M, Grimm D. Biomarkers for anti-angiogenic therapy in cancer. Int J Mol Sci. 2013;14: 9338–9364. doi: 10.3390/ijms14059338 23629668 10.3390/ijms14059338 PMC3676786

[27] Finley SD, Dhar M, Popel AS. Compartment model predicts VEGF secretion and investigates the effects of VEGF trap in tumor-bearing mice. Front Oncol. 2013;3: 196 doi: 10.3389/fonc.2013.00196 23908970 10.3389/fonc.2013.00196 PMC3727077

[28] Finley SD, Dhar M, Popel AS. Compartment model predicts VEGF secretion and investigates the effects of VEGF Trap in tumor-bearing mice. Front Oncol. 2013;3: 196 doi: 10.3389/fonc.2013.00196 23908970 10.3389/fonc.2013.00196 PMC3727077

[29] Finley SD, Popel AS. Effect of tumor microenvironment on tumor VEGF during anti-VEGF treatment: systems biology predictions. J Natl Cancer Inst. 2013;105: 802–811. doi: 10.1093/jnci/djt093 23670728 10.1093/jnci/djt093 PMC3672077

[30] Yen P, Finley SD, Engel-Stefanini MO, Popel AS. A two-compartment model of VEGF distribution in the mouse. PLoS One. 2011;6: e27514 doi: 10.1371/journal.pone.0027514 22087332 10.1371/journal.pone.0027514 PMC3210788

[31] Finley SD, Popel AS. Predicting the effects of anti-angiogenic agents targeting specific VEGF isoforms. AAPS J. 2012;14: 500–509. doi: 10.1208/s12248-012-9363-4 22547351 10.1208/s12248-012-9363-4 PMC3385824

[32] Finley SD, Engel-Stefanini MO, Imoukhuede PI, Popel AS. Pharmacokinetics and pharmacodynamics of VEGF-neutralizing antibodies. BMC Syst Biol. 2011;5: 193 doi: 10.1186/1752-0509-5-193 22104283 10.1186/1752-0509-5-193 PMC3229549

[33] Simeoni M, Magni P, Cammia C, Nicolao G De, Croci V, Pesenti E, et al Predictive Pharmacokinetic-Pharmacodynamic Modeling of Tumor Growth Kinetics in Xenograft Models after Administration of Anticancer Agents Predictive Pharmacokinetic-Pharmacodynamic Modeling of Tumor Growth Kinetics in Xenograft Models after Administratio. Cancer Res. 2004;64: 1094–1101. doi: 10.1158/0008-5472.CAN-03-2524 14871843 10.1158/0008-5472.can-03-2524

[34] Roland CL, Dineen SP, Lynn KD, Sullivan LA, Dellinger MT, Sadegh L, et al Inhibition of vascular endothelial growth factor reduces angiogenesis and modulates immune cell infiltration of orthotopic breast cancer xenografts. Mol Cancer Ther. 2009;8: 1761–1771. doi: 10.1158/1535-7163.MCT-09-0280 19567820 10.1158/1535-7163.MCT-09-0280

[35] Zibara K, Awada Z, Dib L, El-Saghir J, Al-Ghadban S, Ibrik A, et al Anti-angiogenesis therapy and gap junction inhibition reduce MDA-MB-231 breast cancer cell invasion and metastasis in vitro and in vivo. Sci Rep. Nature Publishing Group; 2015;5: 12598 doi: 10.1038/srep12598 26218768 10.1038/srep12598 PMC4517444

[36] Tan G, Kasuya H, Sahin TT, Yamamura K, Wu Z, Koide Y, et al Combination therapy of oncolytic herpes simplex virus HF10 and bevacizumab against experimental model of human breast carcinoma xenograft. Int J Cancer. 2015;136: 1718–1730. doi: 10.1002/ijc.29163 25156870 10.1002/ijc.29163

[37] Volk LD, Flister MJ, Bivens CM, Stutzman A, Desai N, Trieu V, et al Nab-paclitaxel efficacy in the orthotopic model of human breast cancer is significantly enhanced by concurrent anti-vascular endothelial growth factor A therapy. Neoplasia. 2008;10: 613–623. doi: 10.1593/neo.08302 18516298 10.1593/neo.08302 PMC2386546

[38] Volk LD, Flister MJ, Chihade D, Desai N, Trieu V, Ran S. Synergy of nab-paclitaxel and bevacizumab in eradicating large orthotopic breast tumors and preexisting metastases. Neoplasia. 2011;13: 327–338. doi: 10.1593/neo.101490 21472137 10.1593/neo.101490 PMC3071081

[39] Rohrs JA, Sulistio CD, Finley SD. Predictive model of thrombospondin-1 and vascular endothelial growth factor in breast tumor tissue. Npj Syst Biol Appl. 2016;2: 16030 doi: 10.1038/npjsba.2016.30 28713587 10.1038/npjsba.2016.30 PMC5507330

[40] Rohrs JA, Wang P, Finley SD. Predictive model of lymphocyte-specific protein tyrosine kinase (LCK) autoregulation. Cell Mol Bioeng. 2016;9: 351–367. doi: 10.1007/s12195-016-0438-7 27547268 10.1007/s12195-016-0438-7 PMC4978775

[41] Finley SD, Gupta D, Cheng N, Klinke DJ. Inferring relevant control mechanisms for interleukin-12 signaling in naive CD4+ T cells. Immunol Cell Biol. Nature Publishing Group; 2011;89: 100–110. doi: 10.1038/icb.2010.69 20479776 10.1038/icb.2010.69 PMC3004982

[42] Lignet F, Benzekry S, Wilson S, Billy F, Saut O, Tod M, et al Theoretical investigation of the efficacy of antiangiogenic drugs combined to chemotherapy in xenografted mice. J Theor Biol. 2013;320: 86–99. doi: 10.1016/j.jtbi.2012.12.013 23261980 10.1016/j.jtbi.2012.12.013

[43] Jubb AM, Miller KD, Rugo HS, Harris AL, Chen D, Reimann JD, et al Impact of exploratory biomarkers on the treatment effect of bevacizumab in metastatic breast cancer. Clin Cancer Res. 2011;17: 372–381. doi: 10.1158/1078-0432.CCR-10-1791 21224365 10.1158/1078-0432.CCR-10-1791 PMC3023787

[44] Willett CG, Boucher Y, di Tomaso E, Duda DG, Munn LL, Tong RT, et al Direct evidence that the VEGF-specific antibody bevacizumab has antivascular effects in human rectal cancer. Nat Med. 2004;10: 145–147. doi: 10.1038/nm988 14745444 10.1038/nm988 PMC2693485

[45] Jain RK. Normalizing tumor microenvironment to treat cancer: bench to bedside to biomarkers. J Clin Oncol. 2013;31: 2205–2218. doi: 10.1200/JCO.2012.46.3653 23669226 10.1200/JCO.2012.46.3653 PMC3731977

[46] Enderling H, Chaplain MAJ. Mathematical Modeling of Tumor Growth and Treatment. Current Pharmaceutical Design. 2014 pp. 4934–4940. doi: http://dx.doi.org/10.2174/1381612819666131125150434 24283955 10.2174/1381612819666131125150434

[47] Hahnfeldt P, Panigrahy D, Folkman J, Hlatky L. Tumor Development under Angiogenic Signaling. Cancer Res. 1999;59: 4770 LP–4775. 10519381

[48] Benzekry S, Lamont C, Beheshti A, Tracz A, Ebos JML, Hlatky L, et al Classical Mathematical Models for Description and Prediction of Experimental Tumor Growth. PLOS Comput Biol. Public Library of Science; 2014;10: e1003800 doi: 10.1371/journal.pcbi.1003800 25167199 10.1371/journal.pcbi.1003800 PMC4148196

[49] Lewin M, Bredow S, Sergeyev N, Marecos E, Bogdanov A Jr., Weissleder R. In vivo assessment of vascular endothelial growth factor-induced angiogenesis. Int J Cancer. 1999;83: 798–802. 10597197 10.1002/(sici)1097-0215(19991210)83:6<798::aid-ijc16>3.0.co;2-w

[50] Bogin L, Margalit R, Mispelter J, Degani H. Parametric imaging of tumor perfusion using flow- and permeability-limited tracers. J Magn Reson Imaging. 2002;16: 289–299. doi: 10.1002/jmri.10159 12205585 10.1002/jmri.10159

[51] Cao M, Liang Y, Shen C, Miller KD, Stantz KM. Developing DCE-CT to Quantify Intra-Tumor Heterogeneity in Breast Tumors With Differing Angiogenic Phenotype. IEEE Trans Med Imaging. 2009;28: 861–871. doi: 10.1109/TMI.2008.2012035 19150783 10.1109/TMI.2008.2012035

[52] Christensen J, Vonwil D, Prasad VS. Non-invasive in vivo imaging and quantification of tumor growth and metastasis in rats using cells expressing far-red fluorescence protein. PLoS One. 2015;10: 1–14. doi: 10.1371/journal.pone.0132725 26186005 10.1371/journal.pone.0132725 PMC4505884

[53] Yang J, Wang X, Fuh G, Yu L, Wakshull E, Khosraviani M, et al Comparison of binding characteristics and in vitro activities of three inhibitors of vascular endothelial growth factor A. Mol Pharm. 2014;11: 3421–3430. doi: 10.1021/mp500160v 25162961 10.1021/mp500160v

[54] Kreeger PK. Using partial least squares regression to analyze cellular response data. Sci Signal. 2013;6: tr7 doi: 10.1126/scisignal.2003849 23592846 10.1126/scisignal.2003849 PMC4965874

[55] Prasasya RD, Vang KZ, Kreeger PK. A Multivariate Model of ErbB Network Composition Predicts Ovarian Cancer Cell Response to Canertinib. Biotechnol Bioeng. 2012;109: 213–224. doi: 10.1002/bit.23297 21830205 10.1002/bit.23297 PMC3786202

[56] Geladi P, Kowalski BR. Partial least-squares regression: a tutorial. Anal Chim Acta. 1986;185: 1–17.

