# Supplementary figures and images for: Mechanistic modeling quantifies the influence of tumor growth kinetics on the response to anti-angiogenic treatment
Source: PLoS Comput Biol. 2017 Dec 21;13(12):e1005874. doi: 10.1371/journal.pcbi.1005874 (PMC5739350; doi:10.1371/journal.pcbi.1005874)

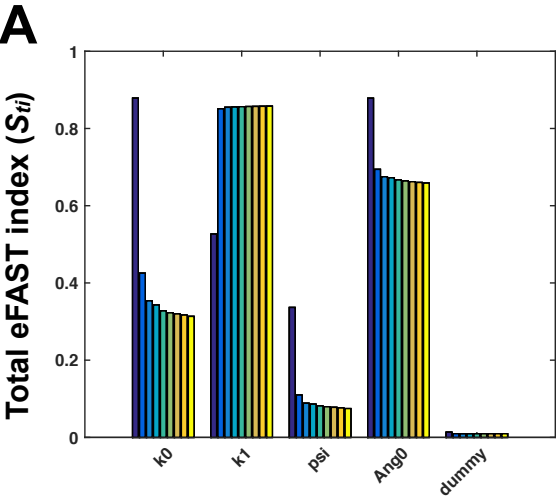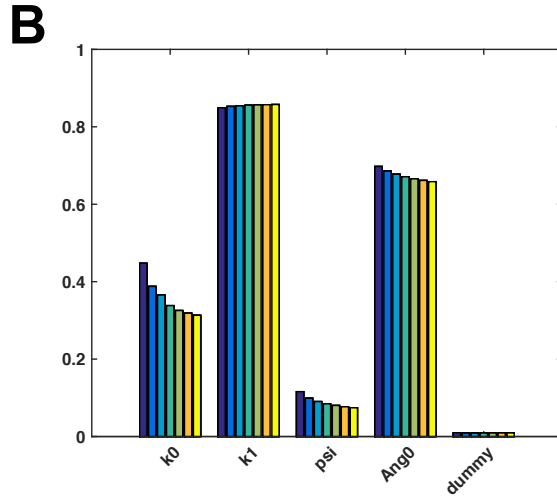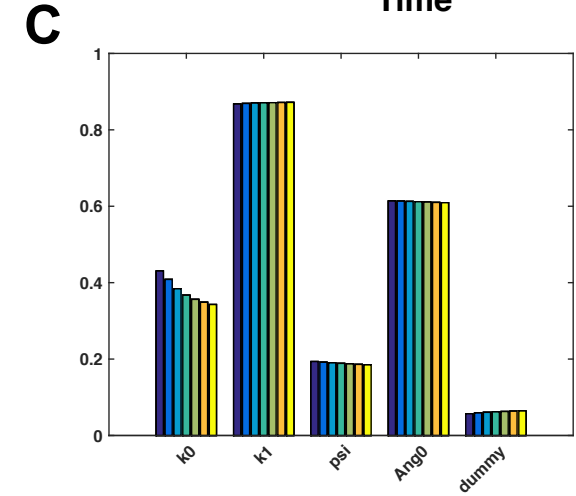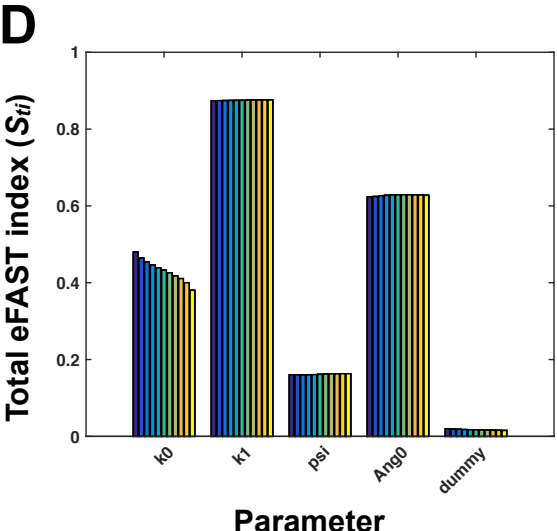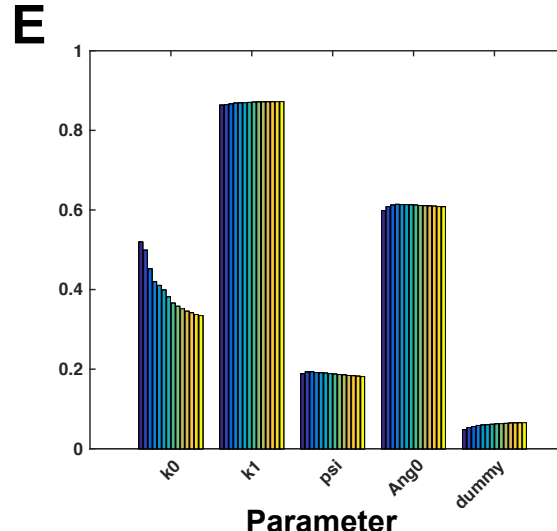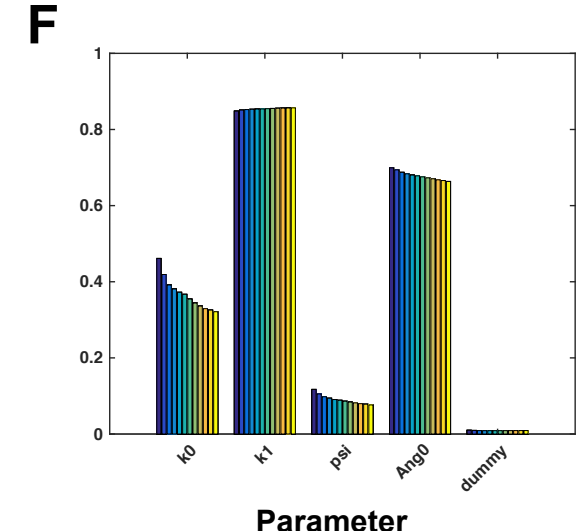

Supplement: S1 Fig — The sensitivity indices estimated using the extended Fourier Amplitude Sensitivity Test (eFAST) quantifying the variance in the model output (tumor volume without treatment) with respect with covariances in combinations of model inputs: the tumor growth parameters k0, k1, ѱ, and Ang0 at distinct times for each dataset. A, Roland [34]. B, Zibara [35]. C, Tan [36]. D, Volk 2008 [37]. E, Volk 2011a [38]. F, Volk 2011b [38]. The sensitivity indices for the growth parameters are compared to a dummy variable that is not included in the model. Indices that are significantly different from the dummy variable influence the model output. We used a cutoff of 0.4 to select which parameters to fit in the parameter estimation. (PDF) [file pcbi.1005874.s001.pdf]
