# Supplementary material for: Mechanistic modeling quantifies the influence of tumor growth kinetics on the response to anti-angiogenic treatment
Source: PLoS Comput Biol. 2017 Dec 21;13(12):e1005874. doi: 10.1371/journal.pcbi.1005874 (PMC5739350; doi:10.1371/journal.pcbi.1005874)
Supplement: S5 Table — This table presents the equations for how the relative volume of the interstitial space changes as a function of the total tumor volume. This equation is unique for each of the datasets investigated. (PDF) [file pcbi.1005874.s006.pdf]

S5 Table. Equations describing change in relative volume of the interstitial space

Dataset	Relative volume of interstitial space (cm^3/cm^3 tissue)
Roland	$Vol_{IS} = 0.8323 \cdot e^{(-0.239 \cdot V(t))}$
Zibara	$Vol_{IS} = 0.8247 \cdot e^{(-0.069 \cdot V(t))}$
Tan	$Vol_{IS} = 0.8343 \cdot e^{(-0.062 \cdot V(t))}$
Volk (2008)	$Vol_{IS} = 0.8628 \cdot e^{(-0.068 \cdot V(t))}$
Volk (2011a)	$Vol_{IS} = 0.8557 \cdot e^{(-0.081 \cdot V(t))}$
Volk (2011b)	$Vol_{IS} = 0.8536 \cdot e^{(-0.068 \cdot V(t))}$
