# Supplementary material for: Mechanistic modeling quantifies the influence of tumor growth kinetics on the response to anti-angiogenic treatment
Source: PLoS Comput Biol. 2017 Dec 21;13(12):e1005874. doi: 10.1371/journal.pcbi.1005874 (PMC5739350; doi:10.1371/journal.pcbi.1005874)
Supplement: S1 Dataset — This file contains a description of the three-compartment computational model, including parameter values and initial conditions. (PDF) [file pcbi.1005874.s007.pdf]

S1 Dataset – Detailed description of computational model

Parameters

Geometry. The geometric parameters for the tumor compartment are summarized in **Table S1**. The tumor cell diameter is assumed to be that of MCF-7 breast tumor cells, 12 mm [1]. Assuming tumor cells are dodecahedral, rather than exactly spherical, we set the tumor cell volume and surface area to be 497 mm^3 and 452 mm^2, respectively. Based on the average luminal diameter of capillaries in growing MCF-7 xenografts, 13.94 mm [2-4], an endothelial cell thickness of 0.5 mm, and the relationship between total perimeter and total cross-sectional area in breast cancer capillaries [5, 6], we estimate the capillary perimeter to be 57.7 mm.

We take the extracellular fluid volume fraction in breast tumor xenografts to be 45%, based on a range of measurements, 33% - 76% [1, 7]. . This volume fraction is divided into interstitial space and intravascular space. Using the capillary dimensions described above and an intravascular volume of 10% [8-10], the capillary density is calculated to be 655 capillaries/mm^2. Based on a cell thickness of 0.5 mm, the volume occupied by the endothelial cells of the microvessels is 1.5%. Cancer cells occupy the remaining tissue volume of 53.5%. The volume fractions of microvessels and tumor cells are then used to calculate the total surface area of all vessels and tumor cells per unit volume of tissue: 378 cm^2 endothelial cell surface / cm^3 tissue and 2939 cm^2 tumor cell surface / cm^3 tissue.

The interstitial space is composed of extracellular matrix (ECM), and basement membranes associated with the microvessels (endothelial basement membrane, EBM) and tumor cells (parenchymal basement membrane, PBM). The thickness of the basement membranes is assumed to be 50 nm and 30 nm, for the EBM and PBM, respectively, yielding volume fractions of 0.0081 and 0.0015 cm^3 / cm^3 tissue. The remaining volume of the interstitial space is the ECM volume (34.04%).

Each region of the interstitial space is represented as a porous medium that contains a solid fraction composed primarily of collagen that is unavailable to VEGF, and a fluid fraction that is accessible to VEGF. The size of the pores further limits the volume available for VEGF to diffuse. Therefore, the available volume in the ECM and basement membranes is calculated as the product of the volume, fluid fraction, and partition coefficient. The fluid fraction is the non-collagen fraction and is calculated by using the total collagen content in interstitial space. Given limited data for this measurement, we used 5%, the same value as in our previous models [11-14]. The ratio of basement membrane collagen to total body collagen is assumed to be 0.3, which yields 0.0482 for the ratio of ECM collagen to total body collagen. The fluid fractions are then 0.7 for the basement membranes and 0.9318 for the ECM. The partition coefficient is the ratio of available fluid volume to interstitial fluid volume. We take 0.9 for the partition coefficient for the EBM [15], and the same value is used for the ECM and PBM, as it is difficult to distinguish basement membranes and the ECM [16]. The available fluid volume for the ECM, EBM, and PBM are therefore 0.2916, 9.720×10^{-4}, and 5.082×10^{-3} cm^3 / cm^3 tissue, respectively.

Initial concentrations. Receptor densities and ECM binding site densities are listed in **Table S2**. VEGFR1, VEGFR2, and NRP1 on the luminal and abluminal surfaces of diseased endothelial cell surfaces and on tumor cells are based on quantitative flow cytometry measurements in endothelial cells isolated from tumor tissue, as described in [13]. We assume NRP2 surface concentration on tumor cells at the same level as NRP1. The initial concentrations of all other species are zero.

Kinetic parameters. The kinetic rates for VEGF binding to and dissociating from receptors, co-receptors, and glycosaminoglycan (GAG) chains in the ECM and basement membranes are the same as in our previous papers, based on experimental data [11-13, 17] and are given in **Table S3**. We use experimental data from [18] for the on and off rates of VEGF binding to the anti-VEGF agent, bevacizumab.

Intercompartmental transport. Transport parameters for VEGF, anti-VEGF and the VEGF/anti-VEGF complex are listed in **Table S4**. Parameters that govern transport between the normal and blood compartments are the same as in our previous models [14, 17].

Secretion rates of soluble species. Tumor cells secrete VEGF into the tumor interstitium at a ratio of 50:50 for $VEGF_{121}$:$VEGF_{165}$, based on experimental quantification of mRNA isoform expression levels [19-23]. Here, we also consider VEGF secretion by EC. We set the secretion ratio of $VEGF_{120}$:$VEGF_{164}$ by EC to be 10:90, similar to the isoform ratio in muscle tissue, since to our knowledge, this ratio has not been determined experimentally. Additionally, we assume normal and tumor EC secrete the same amount of VEGF; tumor EC are a small fraction of the total EC in the body, thus this assumption should not affect VEGF distribution. In our previous work [14], we fit the rates of VEGF secretion by muscle fibers, EC, and tumor cells by parameter optimization, fitting to experimental data from Rudge and coworkers [24]. These fitted values are used in the current model.

The model also includes soluble factors sVEGFR1 and a2M. Endothelial cells are a source of sVEGFR1; therefore, sVEGFR1 is secreted in all three compartments. Endothelial cells also secrete a2M; however, due to its large size, a2M is not transported via transendothelial macromolecular permeability and is confined to the blood compartment. The rates of secretion of sVEGFR1 and a2M are given in Table S4 (below).

Molecular species are removed from the system via two mechanisms: plasma clearance and proteolytic degradation. The values of these parameters are in **Table S4**. For the normal endothelium, the permeability to sVEGFR1 and VEGF/sVEGFR1 is calculated using an empirical relation between the Stokes-Einstein radius, $a_{E,}$ and molecular weight ($a_E = 0.483\times (MW)^{0.386}$) , the corresponding theoretical macromolecular permeability-surface area product, *PS* [25], and the capillary surface area, *S*. Taking microvascular permeability as *PS/S*, and the calculated value is on the order of 10^{-8} cm/s, between the normal and blood compartments. Since tumor vasculature is more permeable than normal microvessels [26], we assume that the microvascular permeability between the tumor and blood is an order of magnitude higher than permeability between normal and blood for both VEGF and the anti-VEGF or complex. Therefore, the permeability to VEGF is 4×10^{-7} cm/s and 3×10^{-7} cm/s for the anti-VEGF and VEGF/anti-VEGF complex. The permeability to sVEGFR1 and VEGF-bound to sVEGFR1 is 1.5×10^{-7} cm/s.

Supplementary Tables

Table S1. Geometric parameters

	Value	Units	Reference
**Cancer cells**			
Tumor cell external diameter	12	μm	[1]
Volume of one cell	905	μm^3	Calculated, see text
Surface area of one cell	497	μm^2	Calculated, see text
**Microvessels**			
Average luminal diameter	13.9	μm	[2]
Endothelial cell thickness	0.5	μm	Based on normal microvessels [27]
Average external diameter	14.9	μm	Calculated, see text
Cross sectional area of one vessel	175.3	μm^2	Calculated, see text
Perimeter of one vessel	57.7	μm	Calculated, see text
Capillary density	655	capillaries/mm^2	Calculated, see text
**Volume fractions**			
Interstitial space	35.0%	cm^2/cm^3 tissue	Based on [1, 7]
Cancer cells	53.5%	cm^2/cm^3 tissue	Calculated, see text
Microvessels	11.5%	cm^2/cm^3 tissue	Calculated, see text
of which intravascular space	10.0%	cm^2/cm^3 tissue	Based on [8-10]
**Surface areas**			
Tumor cells	2939	cm^2/cm^3 tissue	Calculated, see text
Microvessels	378	cm^2/cm^3 tissue	Calculated, see text
**Basement membranes (BM)**			
Thickness of tumor cell BM	30	nm	Based on [28]
Basement membrane volume (tumor cells)	0.00807	cm^3/cm^3	Calculated, see text
of which available to VEGF	0.00508	cm^3/cm^3 tissue	Calculated, see text
Thickness of microvessel BM	50	nm	Based on [28]
Basement membrane volume (microvessels)	0.00154	cm^3/cm^3 tissue	Calculated, see text
of which available to VEGF	0.000972	cm^3/cm^3 tissue	Calculated, see text
Extracellular matrix volume	0.3375	cm^3/cm^3 tissue	Calculated, see text
of which available to VEGF	0.2892	cm^3/cm^3 tissue	Calculated, see text

Table S2. Initial concentrations

Normal compartment

	Value	Units
**VEGFR-1**		
Abluminal EC	0	dimers/EC
Muscle fibers	0	dimers/fiber
**VEGFR-2**		
Abluminal EC	0	dimers/EC
Muscle fibers	0	dimers/ fiber
**NRP-1**		
Abluminal EC	39748	dimers/EC
Muscle fibers	39500	dimers/fiber
**NRP-2**		
Abluminal EC	0	dimers/EC
Muscle fibers	0	dimers/fiber
**ECM binding density**	0.75	μM
**EBM binding density**	13	μM
**PBM binding density**	13	μM

Blood compartment

	Value	Units
**VEGFR-1**		
Luminal EC (normal)	3750	dimers/EC
Luminal EC (diseased)	3750	dimers/EC
**VEGFR-2**		
Luminal EC (normal)	3750	dimers/EC
Luminal EC (diseased)	3750	dimers/EC
**NRP-1**		
Luminal EC (normal)	3750	dimers/EC
Luminal EC (diseased)	3750	dimers/EC
**NRP-2**		
Luminal EC (normal)	0	dimers/EC
Luminal EC (diseased)	0	dimers/EC
**ECM binding density**	0	μM
**EBM binding density**	0	μM
**PBM binding density**	0	μM

Tumor compartment

	Value	Units
**VEGFR-1**		
Abluminal EC	3750	dimers/EC
Tumor cells	1100	dimers/TC
**VEGFR-2**		
Abluminal EC	300	dimers/EC
Tumor cells	550	dimers/ TC
**NRP-1**		
Abluminal EC	39748	dimers/EC
Tumor cells	39500	dimers/TC
**NRP-2**		
Tumor cells	39500	dimers/TC
**ECM binding density**	0.75	μM
**EBM binding density**	13	μM
**PBM binding density**	13	μM

EC = endothelial cell; TC = tumor cell

Table S3. Kinetic parameters

	Value	Unit	Reference
**VEGF binding to VEGFR-1**			
k_{on}	3×10^7	$M^{-1}s^{-1}$	[11, 12]
k_{off}	10^{-3}	s^{-1}	[11, 12]
K_d	33	pM	[11, 12]
**VEGF binding to VEGFR-2**			
k_{on}	10^7	$M^{-1}s^{-1}$	[11, 12]
k_{off}	10^{-3}	s^{-1}	[11, 12]
K_d	100	pM	[11, 12]
**VEGF binding to NRP-1**			
k_{on}	3.2×10^6	$M^{-1}s^{-1}$	[11, 12]
k_{off}	10^{-3}	s^{-1}	[11, 12]
K_d	312.5	pM	[11, 12]
**VEGF binding to GAGs**			
k_{on}	4.20×10^5	$M^{-1}s^{-1}$	[11, 12]
k_{off}	10^{-2}	s^{-1}	[11, 12]
K_d	24	pM	[11, 12]
**Coupling of NRP-1 and VEGFR-1**			
k_c	10^{14}	$(mol/cm^2)^{-1}\,s^{-1}$	[11, 12]
k_{off}	10^{-2}	s^{-1}	[11, 12]
**Coupling of NRP-1 and VEGFR-2**			
$k_{cV165R2,N1}$	3.1×10^{13}	$(mol/cm^2)^{-1}\,s^{-1}$	[11, 12]
$k_{offV165R2,N1}$	10^{-3}	s^{-1}	[11, 12]
$k_{cV165N1,R2}$	10^{14}	$(mol/cm^2)^{-1}\,s^{-1}$	[11, 12]
$k_{offV165N1,R2}$	10^{-3}	s^{-1}	[11, 12]
**VEGFR Internalization**			
k_{int}	2.8×10^{-4}	s^{-1}	[11, 12]
**$VEGF_{121}$ binding to anti-VEGF**			
k_{on}	5.4×10^4	$M^{-1}s^{-1}$	[18]
k_{off}	2.19×10^{-5}	s^{-1}	[18]
K_d	4456	pM	[18]

**VEGF$_{165}$ binding to anti-VEGF**			
k_{on}	5.4×10^{4}	$M^{-1}s^{-1}$	[18]
k_{off}	2.19×10^{-5}	s^{-1}	[18]
K_d	4456	pM	[18]
**VEGF binding to a2M**			
k_{on}	25	$M^{-1}s^{-1}$	Calculated
k_{off}	10^{-4}	s^{-1}	Assumed
K_d	4.0	mM	[29]
**VEGF binding to a2M$_{fast}$**			
k_{on}	2.4×10^{2}	$M^{-1}s^{-1}$	Calculated
k_{off}	10^{-4}	s^{-1}	Assumed
K_d	0.42	mM	[29]
**sVEGFR1 binding to VEGF**			
k_{on}	3×10^{7}	$M^{-1}s^{-1}$	Assumed, based on VEGF binding to VEGFR1
k_{off}	10^{-3}	s^{-1}	Assumed
K_d	33	pM	Assumed
**sVEGFR1 binding to NRP-1**			
k_{on}	5.6×10^{6}	$M^{-1}s^{-1}$	Calculated
k_{off}	10^{-2}	s^{-1}	Assumed, based on VEGFR1 coupling to NRP1
K_d	1.8	nM	[30]
**sVEGFR1 binding to GAGs**			
k_{on}	4.20×10^{5}	$M^{-1}s^{-1}$	Assumed, based on VEGF$_{165}$ binding to GAG
k_{off}	10^{-2}	s^{-1}	Assumed
K_d	24	pM	Assumed

Table S4. Transport parameters

	Value	Unit	Reference
**Permeability between normal and blood**			
VEGF	4.0×10^{-8}	cm/s	[12]
Anti-VEGF & VEGF/anti-VEGF complex	3.0×10^{-8}	cm/s	[12]
Soluble VEGFR1	1.5×10^{-8}	cm/s	Calculated, see text
Soluble VEGFR1/VEGF complex	1.5×10^{-8}	cm/s	Calculated, see text
**Permeability between tumor and blood**			
VEGF	4.0×10^{-7}	cm/s	Assumed, see text
Anti-VEGF & VEGF/anti-VEGF complex	3.0×10^{-7}	cm/s	Assumed, see text
Soluble VEGFR1	1.5×10^{-7}	cm/s	Assumed, see text
Soluble VEGFR1/VEGF complex	1.5×10^{-7}	cm/s	Assumed, see text
**Clearance**			
VEGF	2.3×10^{-1}	min^{-1}	[31]
Anti-VEGF	8.9×10^{-4}	min^{-1}	[17]
VEGF/anti-VEGF complex	2.8×10^{-4}	min^{-1}	[17]
Soluble VEGFR1	3.0×10^{-4}	min^{-1}	[32]
Soluble VEGFR1/VEGF complex	3.0×10^{-4}	min^{-1}	[32]
Alpha-2-macroglobulin (a2M)	2.6×10^{-3}	min^{-1}	[33]
a2M /VEGF complex	2.6×10^{-3}	min^{-1}	Assumed, based on a2M
a2M /VEGF/anti-VEGF complex	2.6×10^{-3}	min^{-1}	Assumed, based on a2M
Activated alpha-2-macroglobulin ($a2M_{fast}$)	2.4×10^{-1}	min^{-1}	[34]
a2M /VEGF complex	2.6×10^{-3}	min^{-1}	Assumed, based on $a2M_{fast}$
**Degradation**			
Soluble VEGFR1	1.2×10^{-2}	min^{-1}	Assumed based on VEGF
Soluble VEGFR1/VEGF complex	1.2×10^{-2}	min^{-1}	Assumed based on VEGF
**Synthesis**			
$VEGF_{164}$ – Normal cells	9.72×10^{-3}	molecules/cell/s	Estimated in [14]
$VEGF_{164}$ – Endothelial cells	6.72×10^{-3}	molecules/cell/s	Estimated in [14]
$VEGF_{164}$ – Tumor cells	0	molecules/cell/s	Tumor cells only secrete human VEGF isoforms

$VEGF_{120}$ – Normal cells	8.45×10^{-4}	molecules/cell/s	Estimated in [14]
$VEGF_{120}$ – Endothelial cells	7.47×10^{-4}	molecules/cell/s	Estimated in [14]
$VEGF_{120}$ – Tumor cells	0	molecules/cell/s	Tumor cells only secrete human VEGF isoforms
$VEGF_{165}$ – Normal cells	0	molecules/cell/s	Only tumor cells secrete human VEGF isoforms
$VEGF_{165}$ – Endothelial cells	0	molecules/cell/s	Only tumor cells secrete human VEGF isoforms
$VEGF_{165}$ – Tumor cells	4.65×10^{-3}	molecules/cell/s	Estimated in [14]
$VEGF_{121}$ – Normal cells	0	molecules/cell/s	Only tumor cells secrete human VEGF isoforms
$VEGF_{121}$ – Endothelial cells	0	molecules/cell/s	Only tumor cells secrete human VEGF isoforms
$VEGF_{121}$ – Tumor cells	4.65×10^{-3}	molecules/cell/s	Estimated in [14]
Alpha-2-macroglobulin	1.8×10^{10}	molecules/cm^3 tissue/s	Calculated, see text
Activated alpha-2-macroglobulin	1.6×10^{10}	molecules/cm^3 tissue/s	Calculated, see text

References

1. Paran Y, Bendel P, Margalit R, Degani H. Water diffusion in the different microenvironments of breast cancer. NMR Biomed. 2004;17(4):170-80.
2. Schaefer C, Schroeder M, Fuhrhop I, Viezens L, Otten J, Fiedler W, et al. Primary tumor dependent inhibition of tumor growth, angiogenesis, and perfusion of secondary breast cancer in bone. J Orth Res. 2011;29(8):1251-8.
3. Kim E, Stamatelos SK, Cebulla J, Bhujwalla ZM, Popel AS, Pathak AP. Multiscale imaging and computational modeling of blood flow in the tumor vasculature. Ann Biomed Eng. 2012;40(11):2425-41.
4. Stamatelos SK, Kim E, Pathak AP, Popel AS. Hybrid bioimage informatics and computational modeling methodology to reconstruct the entire tumor vasculature and evaluate perfusion across regions. Submitted. 2013.
5. Olewniczak S, Chosia M, Kolodziej B, Kwas A, Kram A, Domagala W. Angiogenesis as determined by computerised image analysis and the risk of early relapse in women with invasive ductal breast carcinoma. Pol J Pathol. 2003;54(1):53-9.
6. Olewniczak S, Chosia M, Kwas A, Kram A, Domagala W. Angiogenesis and some prognostic parameters of invasive ductal breast carcinoma in women. Pol J Pathol. 2002;53(4):183-8.
7. Hassid Y, Furman-Haran E, Margalit R, Eilam R, Degani H. Noninvasive magnetic resonance imaging of transport and interstitial fluid pressure in ectopic human lung tumors. Cancer Res. 2006;66:4159-66.
8. Cao M, Liang Y, Shen C, Miller KD, Stantz KM. Developing DCE-CT to quantify intra-tumor heterogeneity in breast tumors with different angiogenic phenotype. IEEE Trans Med Imaging. 2009;28(6):861-71.
9. Bogin L, Margalit R, Mispelter J, Degani H. Parametric imaging of tumor perfusion using flow- and permeability-limited tracers. J Magn Reson Imaging. 2002;16:289-99.
10. Lewin M, Bredow S, Sergeyev N, Marecos E, Bodganov Jr. A, Weissleder R. In vivo assessment of vascular endothelial growth factor-induced angiogenesis. Int J Cancer. 1999;83:798-802.
11. Mac Gabhann F, Popel AS. Targeting neuropilin-1 to inhibit VEGF signaling in cancer: comparison of therapeutic approaches. PLoS Comp Biol. 2006;2(12):e180.
12. Stefanini MO, Wu FTH, Mac Gabhann F, Popel AS. A compartment model of VEGF distribution in blood, healthy and diseased tissues. BMC Syst Biol. 2008;2(1):77.
13. Finley SD, Engel-Stefanini MO, Imoukhuede PI, Popel AS. Pharmacokinetics and pharmacodynamics of VEGF-neutralizing antibodies. BMC Syst Biol. 2011;5:193.
14. Finley SD, Dhar M, Popel AS. Compartment model predicts VEGF secretion and investigates the effects of VEGF Trap in tumor-bearing mice. Front Oncol. 2013;3:196.
15. Yuan F, Krol A, Tong S. Available space and extracellular transport of macromolecules: effects of pore size and connectedness. Ann Biomed Eng. 2001;29(12):1150-8.
16. Hashizume H, Baluk P, Morikawa S, McLean JW, Thurston G, Roberge S, et al. Openings between defective endothelial cells explain tumor vessel leakiness. Am J Pathol. 2000;156(4):1363-80.
17. Yen P, Finley SD, Engel-Stefanini MO, Popel AS. A two-compartment model of VEGF distribution in the mouse. PLoS One. 2011;6(11):e27514.
18. Yang J, Wang X, Fuh G, Yu L, Wakshull E, Khosraviani M, et al. Comparison of Binding Characteristics and In Vitro Activities of Three Inhibitors of Vascular Endothelial Growth Factor A. Mol Pharm. 2014;11(10):3421-30. doi: 10.1021/mp500160v.
19. Stimpfl M, Tong D, Fasching B, Schuster E, Obermair A, Leodolter S, et al. Vascular endothelial growth factor splice variants and their prognostic value in breast and ovarian cancer. Clin Cancer Res. 2002;8(7):2253-9.

20. Yuan A, Yu CJ, Luh KT, Lin FY, Kuo SH, Yang PC. Quantification of VEGF mRNA expression in non-small cell lung cancer using a real-time quantitative reverse transcription-PCR assay and a comparison with quantitative competitive reverse transcription-PCR. Lab Invest. 2000;2000(80):11.
21. Cheung N, Wong MP, Yuen ST, Leung SY, Chung LP. Tissue-specific expression pattern of vascular endothelial growth factor isoforms in the malignant transformation of lung and colon. Hum Pathol. 1998;29(9):910-4.
22. Ljungberg B, Jacobsen J, Haggstrom-Rudolfssson S, Rasmuson T, Lindh G, Grankvist K. Tumor vascular endothelial growth factor (VEGF) mRNA in relation to serum VEGF protein levels and tumour progression in human renal cell carcinoma. Urol Res. 2003;31(5):335-40.
23. Zygalaki E, Tsaroucha EG, Kaklamanis L, Lianidou ES. Quantitative real-time reverse transcription-PCR study of the expression of vascular endothelial growth factor (VEGF) splice variants and VEGF receptors (VEGFR-1 and VEGFR-2) in non-small cell lung cancer. Clin Chem. 2007;53(8):1433-9.
24. Rudge JS, Holash J, Hylton D, Russell M, Jiang S, Leidich R, et al. VEGF Trap complex formation measures production rates of VEGF, providing a biomarker for predicting efficacious angiogenic blockade. Proc Natl Acad Sci U S A. 2007;104(47):18363-70.
25. Garlick DG, Renkin EM. Transport of large molecules from plasma to interstitial fluid and lymph in dogs. Am J Physiol. 1970;219(6):1595-605.
26. Goel S, Duda DG, Xu L, Munn LL, Boucher Y, Fukumura D, et al. Normalization of the vasculature for treatment of cancer and other diseases. Physiol Rev. 2011;91(3):1071-121.
27. Hall JE. The circulation. Guyton and Hall Textbook of Medical Physiology, 12th ed: W.B. Saunders Co; 2011.
28. Baluk P, Morikawa S, Haskell A, Mancuso M, McDonald DM. Abnormalities of basement membrane on blood vessels and endothelial sprouts in tumors. Am J Pathol. 2003;163(5):1801-15.
29. Bhattacharjee G, Asplin IR, Wu SM, Gawdi G, Pizzo SV. The conformation-dependent interaction of alpha-2-macroglobulin with vascular endothelial growth factor. J Biol Chem. 2000;275(35):26806-11.
30. Fuh G, Garcia KC, De Vos AM. The interaction of neuropilin-1 with vascular endothelial growth factor and its receptor flt-1. J Biol Chem. 2000;275:26690-5.
31. Folkman J. Angiogenesis in cancer, vascular rheumatoid and other disease. Nat Med. 1995;1(1):27-31.
32. Wu FTH, Stefanini MO, Mac Gabhann F, Popel AS. A compartment model of VEGF distribution in humans in the presence of soluble VEGF receptor-1 acting as a ligand trap. PLoS One. 2009;4(4):e5108.
33. Hudson NW, Kehoe JM, Koo PH. Mouse alpha-macroglobulin. Structure, function, and a molecular model. Biochem J. 1987;248(3):837-45.
34. Imber MJ, Pizzo SV. Clearance and binding of two electrophoretic “fast” forms of human alpha-2-macroglobulin. J Biol Chem. 1981;256(15):8134-9.
